# Longitudinal changes in the relative toxicity of FDA-approved oncology therapeutics: evidence from paired initial and updated RCT reports

**DOI:** 10.3389/fpubh.2026.1882548

**Published:** 2026-07-06

**Authors:** Weiqiang Song, Jiankun Zhang, Guangpeng Chen, Yunzhao Ji

**Affiliations:** 1 Department of Urology, Hebei Petrochina Central Hospital, Langfang, Hebei, China; 2 School of Public Health, Fudan University, Shanghai, China; 3 Department of Anesthesiology, Hebei Petrochina Central Hospital, Langfang, Hebei, China

**Keywords:** adverse events, comparative toxicity, evidence maturity, FDA approval, oncology drugs, post-marketing surveillance

## Abstract

**Background:**

A growing share of oncology drugs receive FDA approval via expedited pathways based on interim RCT analyses, leaving safety data relatively immature at approval. Whether the relative toxicity of approved therapeutics shifts as trials mature remains unquantified.

**Methods:**

Phase II–III RCTs underpinning FDA oncology approvals from 2006 to 2025 were eligible if both the approval-stage dataset and at least one subsequent publication reported grade ≥3 AEs or serious AEs (SAEs). For each trial, experimental-versus-control odds ratios (ORs) were computed at both timepoints, with within-trial change indexed by the ratio of odds ratios (ROR), defined as the updated OR divided by the initial OR (ROR > 1 indicating greater experimental-arm toxicity after maturation). Random-effects meta-analyses pooled log-scale estimates; meta-regression explored effect modifiers and sources of heterogeneity.

**Results:**

Ninety-nine RCTs met eligibility and, after splitting seven three-arm trials, yielded 106 pairwise comparisons (103 for grade ≥3 AEs; 70 for SAEs). Pooled OR for grade ≥3 AEs rose from 1.09 (95% CI, 0.93–1.22) to 1.29 (1.03–1.43); meta-analytic ROR showed a statistically significant increase at 1.095 (1.038–1.156; *p* = 0.001; *I*^2^ = 12.8%), with 78/103 comparisons shifting toward greater experimental toxicity. For SAEs, pooled OR rose from 1.32 (1.21–1.42) to 1.45 (1.27–1.59); ROR was 1.06 (0.97–1.17; *p* = 0.22; *I*^2^ = 46.8%), directionally concordant but not statistically significant, with upward shifts in 49/70 comparisons. Amplification was most evident for all-cause events and in crossover-permitted, blinded, medium-sized, and later-line trials, and in lung, prostate, and breast cancer. RORs declined across more recent cohorts (*β* = −0.027, *p* = 0.009 for SAEs); larger enrollment, longer follow-up, blinded designs, and all-cause ascertainment predicted greater drift, while mature initial follow-up and metastatic settings attenuated it. We derived an exploratory Toxicity Drift Predictor to estimate post-approval ROR of SAEs from pre-approval trial features.

**Conclusion:**

FDA-approved oncology therapeutics show systematic post-approval increases in relative toxicity, most pronounced for all-cause events and designs prolonging differential exposure. Though attenuated for recent agents, the approval-stage safety profile represents a lower-bound estimate of long-term comparative harm, supporting evidence-maturity–based dynamic oversight with predefined safety-update milestones and structured surveillance of delayed toxicities.

## Introduction

Regulatory authorization of anticancer agents in the United States rests predominantly on pivotal phase II-III randomized controlled trials (RCTs), with the safety database available at approval reflecting the data cut at which the primary efficacy endpoint was first met or a pre-specified interim analysis was triggered. This is true across both regular and expedited approval pathways: in either case, median follow-up at the supporting data cut is typically constrained to 12–36 months, exposure beyond this window is sparse, and late-emerging toxicities have not had time to manifest (1–4). Expedited pathways—accelerated approval, breakthrough therapy designation, priority review, and fast track—further compress this evidentiary window by allowing authorization on the basis of earlier interim analyses and, frequently, surrogate endpoints such as progression-free survival or objective response rate (1, 2); however, the underlying issue of immature safety ascertainment at the approval-supporting data cut is a general property of pivotal-RCT-based authorization rather than a feature unique to expedited routes. The benefit–risk portrait informing approval is therefore, by design, a snapshot of an immature dataset rather than a depiction of the drug’s long-term tolerability (5).

This limitation is particularly consequential given the shift from cytotoxic monotherapy toward immune checkpoint inhibitors, antibody–drug conjugates, bispecific antibodies, targeted kinase inhibitors, and multi-agent combinations (6). Unlike conventional chemotherapy—whose toxicities are largely acute, mechanistically predictable, and reversible after discontinuation—these newer classes generate adverse events (AEs) that are often delayed, cumulative, or chronic (7, 8). Immune-related AEs (irAEs) may first appear months or even years after checkpoint inhibitor initiation and can evolve into permanent endocrinopathies or other organ dysfunction persisting long after therapy ends (9, 10). Multi-targeted kinase inhibitors produce protracted off-target effects tied to sustained pathway inhibition in normal tissues, and combination regimens compound this risk through overlapping and non-additive toxicity mechanisms (11, 12). Because pivotal trials are increasingly reported at the earliest statistically interpretable timepoint, such signals are structurally under-represented in the evidence available to regulators, clinicians, payers, and patients at approval (13).

Existing scrutiny of FDA oncology decisions has concentrated largely on efficacy—whether overall survival benefits are ultimately confirmed for agents approved on surrogate endpoints (14, 15), whether accelerated approvals convert to regular approval within expected timelines (3), or how the evidentiary quality of supporting trials has changed across approval cohorts (1, 16). Safety has been approached less systematically. Existing work has taken two main forms: cross-sectional descriptions of AE frequencies in pivotal trial publications (17), or post-marketing pharmacovigilance analyses of spontaneous-reporting systems, which are limited by selective reporting, missing denominators, and the absence of a randomized comparator (18, 19). What remains largely unexamined is how the relative toxicity of an experimental regimen versus its control evolves as RCT matures from the interim analysis supporting approval to later data cuts.

The clinical and regulatory stakes of this question are concrete. If experimental arms systematically accrue a greater relative burden of severe AEs as trials mature—while control arms, composed largely of shorter-course chemotherapy, placebo, or supportive care, do not—the benefit–risk assumptions that justified approval may require periodic recalibration, predefined safety-update milestones, and intensified surveillance of delayed toxicities (20, 21). If, conversely, relative tolerability remains broadly stable with longer exposure, current reliance on comparatively immature safety databases at approval would be empirically supported. Evidence capable of discriminating between these scenarios across drug classes, tumor types, and approval periods is currently lacking.

To close this gap, we systematically reviewed all phase II–III RCTs supporting FDA oncology approvals from January 2006 to September 2025 that had reported both the initial safety data considered at the time of approval and at least one subsequent update from the same trial. We quantified how the risk of relative AEs associated with FDA-approved oncology therapeutics changed with extended follow-up, and examined trial-level factors associated with the magnitude and direction of these changes. The findings are intended to inform regulatory oversight informed by evidence maturity, post-marketing safety monitoring, and long-term clinical management of patients receiving FDA-approved oncology therapeutics.

## Methods

**Figure 1:**
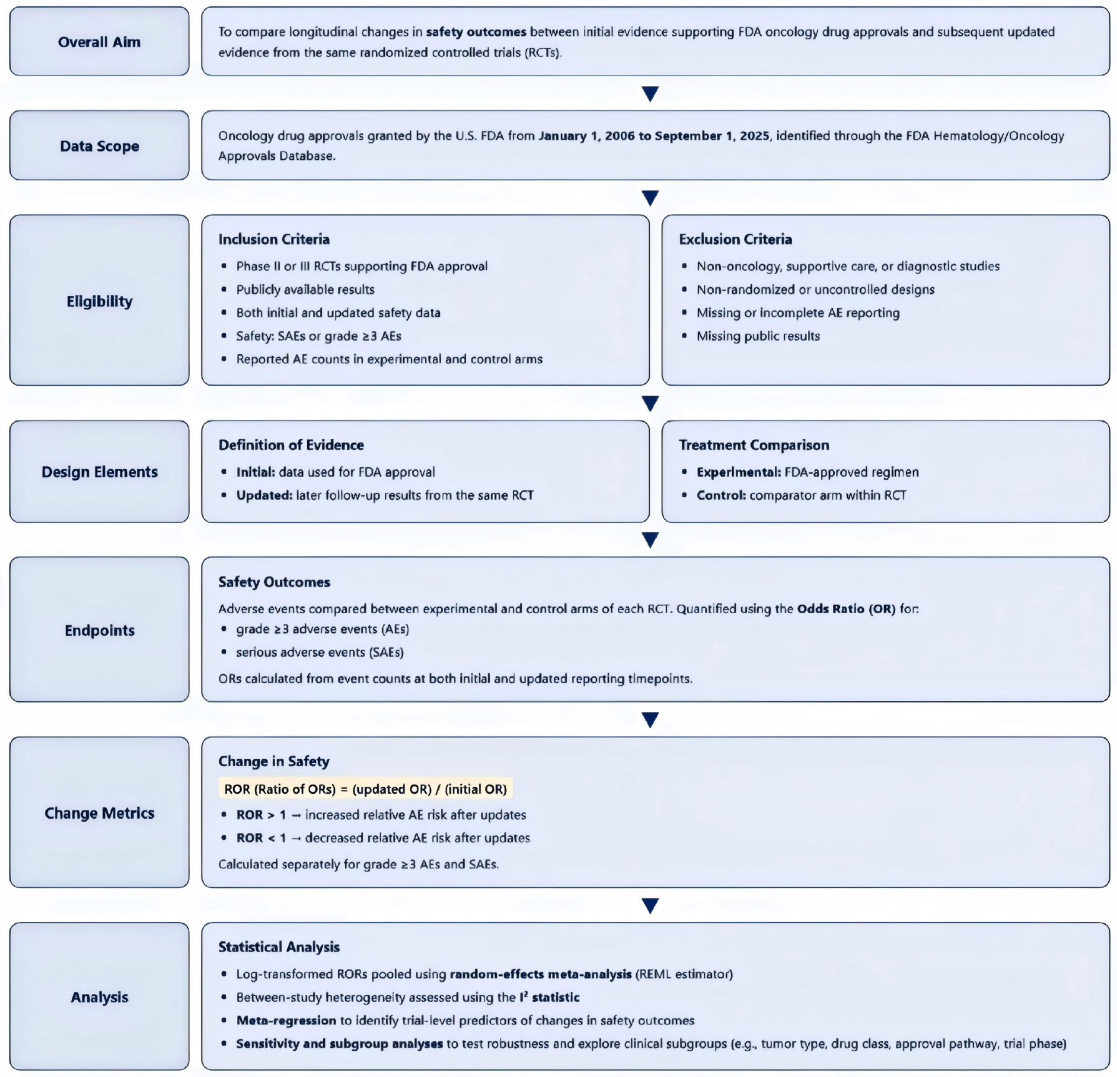
Study process flowchart. AE, adverse event; OR, odds ratio; RCT, randomized controlled trial; ROR, Ratio of updated to initial odds ratios; SAE, serious adverse event.

Overall study process is outlined in Figure 1.

### Sample identification

Oncology drug approvals issued by the US FDA from January 1, 2006 through September 1, 2025 were retrieved from the FDA Hematology/Oncology Approvals Database. To be eligible, a trial had to be a phase II or Ш RCT that served as a basis for one of these approvals and had to have generated at least two rounds of published evidence from the same study: the dataset available to the FDA at approval (hereafter “initial evidence”) and at least one updated analysis of the same trial (“updated evidence”). The updated evidence for every included trial was drawn exclusively from extended follow-up of the same randomized cohort, conducted under the original protocol or its pre-specified amendments. Both regular and accelerated approvals were eligible. Safety endpoints of interest were grade ≥3 AEs and serious AEs (SAEs), as defined by the Common Terminology Criteria for Adverse Events (22). To be included, a trial had to report at least one of these two endpoints in both the initial and the updated publication. Approvals outside oncology, as well as those for supportive care or diagnostic indications, were not considered, and single-arm, non-randomized, or uncontrolled studies were excluded given the absence of a concurrent comparator against which relative toxicity could be quantified.

Supporting publications for each eligible trial were located through MEDLINE and ClinicalTrials.gov, cross-referenced by NCT identifier, generic drug name, and approved indication. For trials with several post-approval data cuts, the most recent publication was retained as the updated evidence, on the premise that it reflects the longest cumulative exposure and therefore the most complete safety ascertainment. Record screening was carried out in duplicate by two reviewers (WS and JZ), and any discrepancy was adjudicated through discussion until agreement was reached.

### Data extraction

Data extraction was performed independently by four authors (WS, GC, JZ and YJ). For each eligible trial, we abstracted information on study design (trial phase, blinding, and whether crossover to the experimental arm was permitted upon progression), the FDA approval date for the relevant indication, the treatment comparison, cancer type, randomized sample size, and follow-up duration reported in each publication. From both the initial and updated publications of the same trial, the number of patients experiencing SAEs and grade ≥3 AEs, together with the corresponding denominator in each arm, were extracted verbatim as reported. For three-arm RCTs, the trial was split into two pairwise comparisons against the common control arm, each treated as a separate unit of analysis. To minimize transcription and interpretation error, every trial was extracted into a structured template and cross-checked by a second reviewer; discrepancies were resolved through re-inspection of the source publication.

### Assessment of changes in relative safety

In our study, the safety endpoints included grade ≥3 AEs and SAE (23). In each RCT, innovative therapy that received FDA approval was defined as the experimental arm, whereas the comparator regimen was defined as the control arm. Safety outcomes were assessed by comparing the odds of experiencing AEs between experimental and control arms, expressed as odds ratios (ORs). ORs were calculated based on number of patients with and without AEs in each arm (24). Changes in relative safety were evaluated using the ratio of odds ratios (ROR), explicitly defined as the OR derived from the updated dataset divided by the OR derived from the initial dataset for the same trial. An ROR > 1 indicated an increased relative risk of AEs in experimental arm after updates.


$$\mathrm{ROR}(\text{ratio in OR})=\frac{\text{OR in updated data}}{\text{OR in initial data}}$$

### Statistical analysis

To quantify how the relative safety profile between experimental and control arms evolved from the initial to the updated data cut, OR of SAEs and of grade ≥3 AEs was computed for each trial at each timepoint. Analyses were carried out on the natural-log scale: for every trial, log-ORs and their standard errors were derived from the extracted event counts and arm-level denominators, and the change between timepoints was obtained by contrasting the updated log-OR against the initial log-OR, with the variance of this contrast approximated via the delta method (25). Pooled estimates were generated with random-effects meta-analyses using inverse-variance weighting and between-study variance estimated by restricted maximum likelihood (REML) (26).

Separate pooled log-ORs were produced for the initial dataset, for the updated dataset, and for the within-trial change between the two, with each subsequently back-transformed for interpretation. Heterogeneity across trials was summarized with the *I*^2^ statistic. Drivers of the observed heterogeneity were explored by meta-regression, taking the log-transformed effect size as the dependent variable. Candidate trial-level covariates were specified in advance on the basis of clinical and methodological relevance, and are enumerated in Supplementary Table S1. To guard against overfitting, the 10-studies-per-covariate convention was observed. Each covariate was first examined in a univariable model; those reaching *p* < 0.10 were advanced to a multivariable model, within which multicollinearity was screened by variance inflation factor, with values exceeding five flagged as problematic (27). As a post-hoc exploratory analysis, we used the SAE multivariable meta-regression coefficients to build a Toxicity Drift Predictor (TDP), which outputs a predicted ROR with 95% prediction interval and a four-tier risk grade. Further details are provided in Supplementary Method S1. All analyses were two-sided, performed in R (version 4.2.3), and used the meta and metafor packages; a *p*-value <0.05 was taken as the threshold for statistical significance.

### Subgroup analyses

To examine whether the within-trial shift in relative safety between the initial and the updated data cut differed across clinically and methodologically meaningful strata, a set of subgroup analyses was conducted. Trials were stratified by year of FDA approval, cancer type, line of therapy, nature of the treatment comparison, trial phase, blinding, permissibility of crossover, metastatic versus non-metastatic setting, sample size, and duration of follow-up. The full list of subgroups, together with the clinical or methodological rationale for including each, is provided in Supplementary Table S2. Data and R code supporting this study are publicly available at OSF.^1^

## Results

**Figure 2:**
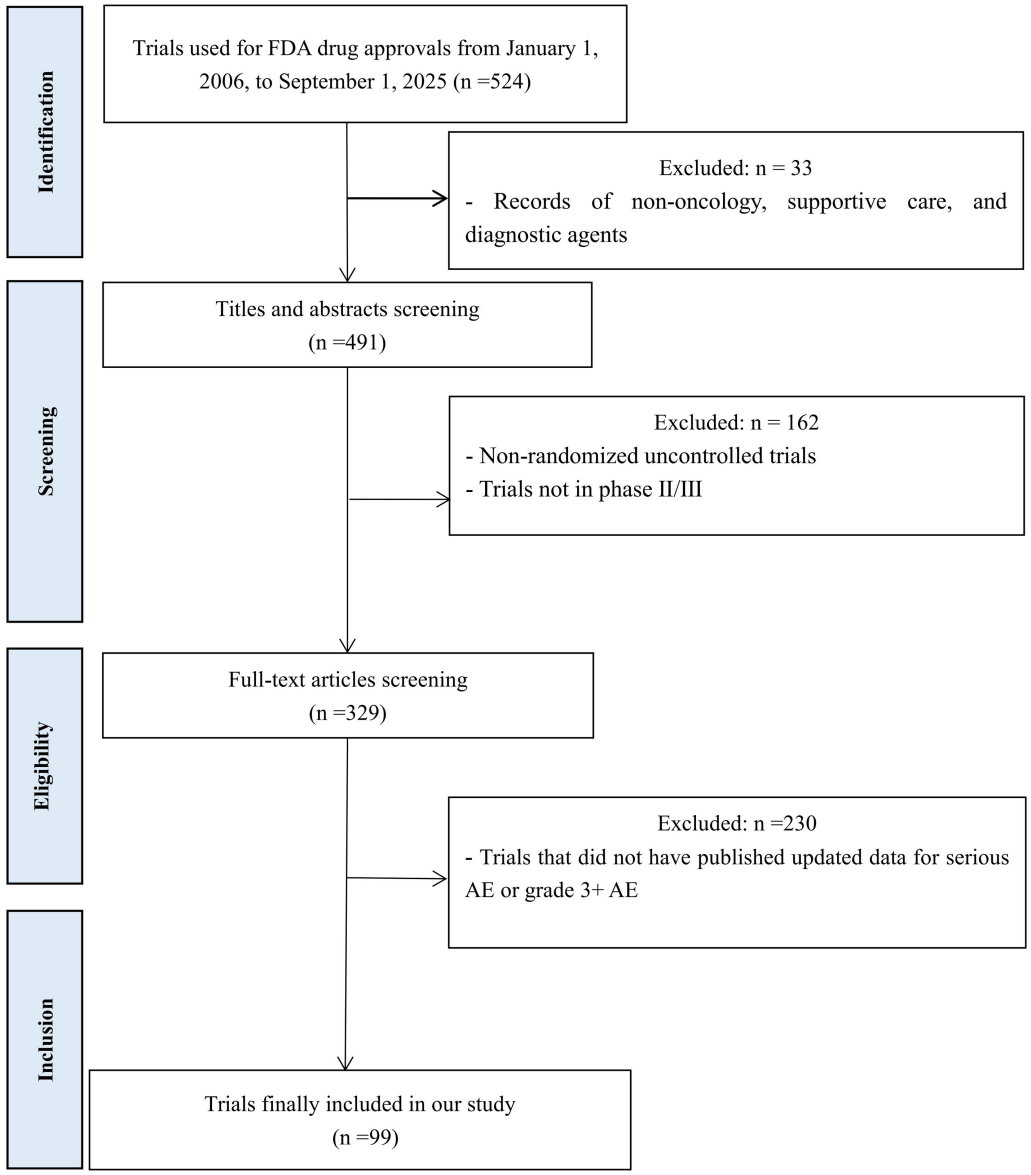
Study flow chart. AE, adverse event.

Study selection process is provided in Figure 2. A total of 524 records were identified through the systematic search, of which 329 RCTs met the eligibility criteria. After exclusion of 230 RCTs for which no subsequent updated publication was available, 99 RCTs (7 three-arm trials) were retained for the final analysis. Reflecting the RCT-based eligibility criteria, 97 of the 99 included trials supported regular FDA approvals, with only BREAKWATER (encorafenib plus cetuximab) and COMBI-d (dabrafenib plus trametinib) supporting accelerated approvals. Because each three-arm trial was split into two pairwise comparisons, the 99 trials yielded 106 pairwise comparisons in total, of which 103 contributed to the grade ≥3 AE analysis and 70 to the SAE analysis. Characteristics of all included trials are summarized in Supplementary Table S3. Dates of FDA approval, initial publication, and updated publication for each trial are provided in Supplementary Table S4. A summary of the systematic search is in Supplementary Tables S5. Subgroup characteristics for each endpoint (grade ≥3 AEs and SAEs) are provided in Supplementary Tables S6, S7.

**Figure 3:**
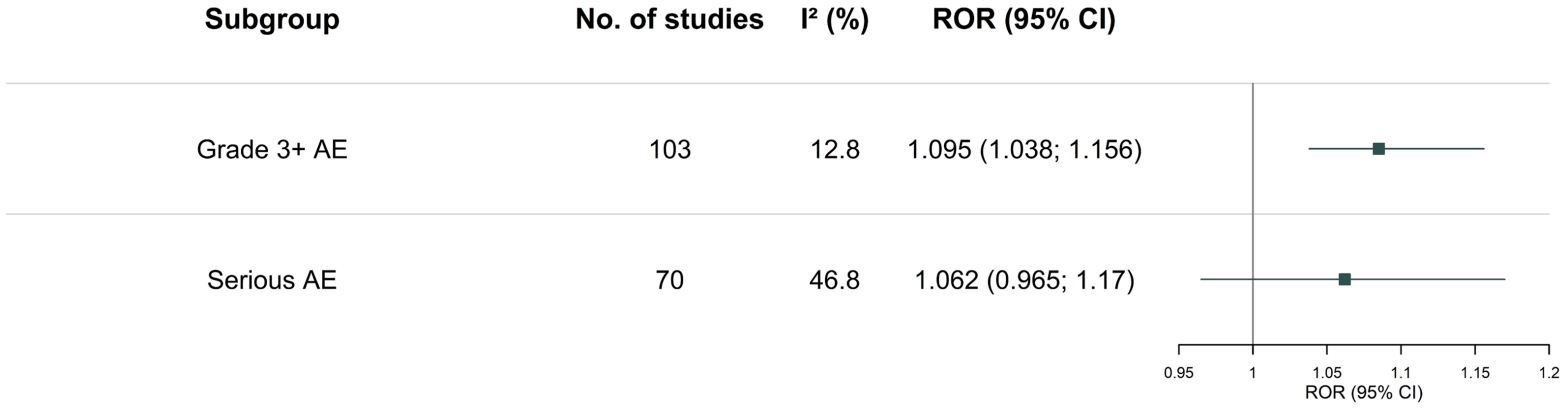
Changes in relative safety of FDA-approved treatments Compared with controls between initial and updated analyses. AE, adverse event; ROR, Ratio of updated to initial odds ratios; SAE, serious adverse event. An ROR > 1 indicates that the relative toxicity of the experimental group compared with the control group became more pronounced after data updates.

**Figure 4:**
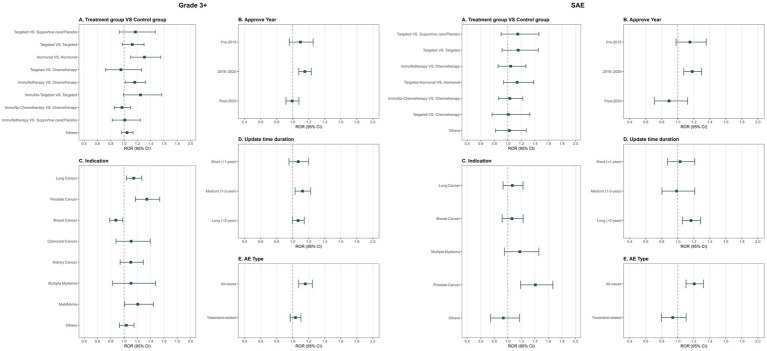
Subgroup analyses of changes between updated and initial relative AE. ROR, ratio of updated to initial odds ratio; AE, adverse event; SAE, serious adverse event. Only key subgroups are presented in this figure. Comprehensive subgroup results are available in Supplementary Table S10; An ROR > 1 indicates that the relative toxicity of the experimental group compared with the control group became more pronounced after data updates.

Pooled ORs for grade ≥3 AEs increased from 1.09 (95% CI, 0.93–1.22) in initial analyses to 1.29 (1.03–1.43) after updates (Supplementary Table S8 for subgroup and heterogeneity details). Among 103 comparisons, 78 showed increased ORs and 25 showed decreases after updates. As shown in Figure 3, updated evidence indicated a consistent increase (*p* = 0.001) in the relative risk of grade ≥3 AEs in experimental arms (meta-analytic ROR, 1.095 [95% CI, 1.038–1.156]; *I*^2^ = 12.8%). All-cause grade ≥3 AEs significantly increased after updates (ROR, 1.16; 1.07–1.25; *p* < 0.001). Treatment-related grade ≥3 AEs showed no statistically significant change after data maturation (ROR, 1.05; 0.97–1.14; *p* = 0.22). This pattern was more apparent in several other subgroups, including subsequent-line treatment (1.177, 1.079–1.284), crossover trials (1.203, 1.092–1.326), blinded studies (1.147, 1.042–1.263), medium-sized trials enrolling 100 to 600 patients (1.141, 1.049–1.242), drugs approved during 2016 to 2020 (1.151, 1.076–1.232), lung cancer (1.147, 1.033–1.274), prostate cancer (1.342, 1.170–1.539), breast cancer (1.206, 1.009–1.443), metastatic settings (1.102, 1.038–1.170), and indications with low or intermediate 5-year survival. More information is in Figure 4 and Supplementary Table S8.

Pooled ORs for SAEs increased from 1.32 (95% CI, 1.21–1.42) in the initial analyses to 1.45 (95% CI, 1.27–1.59) after data updates. Of 70 comparisons, 49 showed increased ORs after updates. The meta-analytic ROR was 1.06 (95% CI, 0.97–1.17; *I*^2^ = 46.8%), indicating no statistically significant overall change (*p* = 0.22) in relative SAE risk after data maturation, although the direction of the point estimate was concordant with the grade ≥3 AE findings. Several subgroups showed significantly increased risk after updates, including subsequent-line treatment, crossover trials, blinded studies, medium-sized trials, drugs approved during 2016–2020, nonmetastatic settings, longer update duration, and all-cause SAEs. These findings were broadly consistent with those for grade ≥3 AEs and suggest a tendency toward higher relative AE risks in experimental arms with longer follow-up. Detailed overall and subgroup results are provided in Figures 3, 4, Supplementary Table S9.

**Figure 5:**
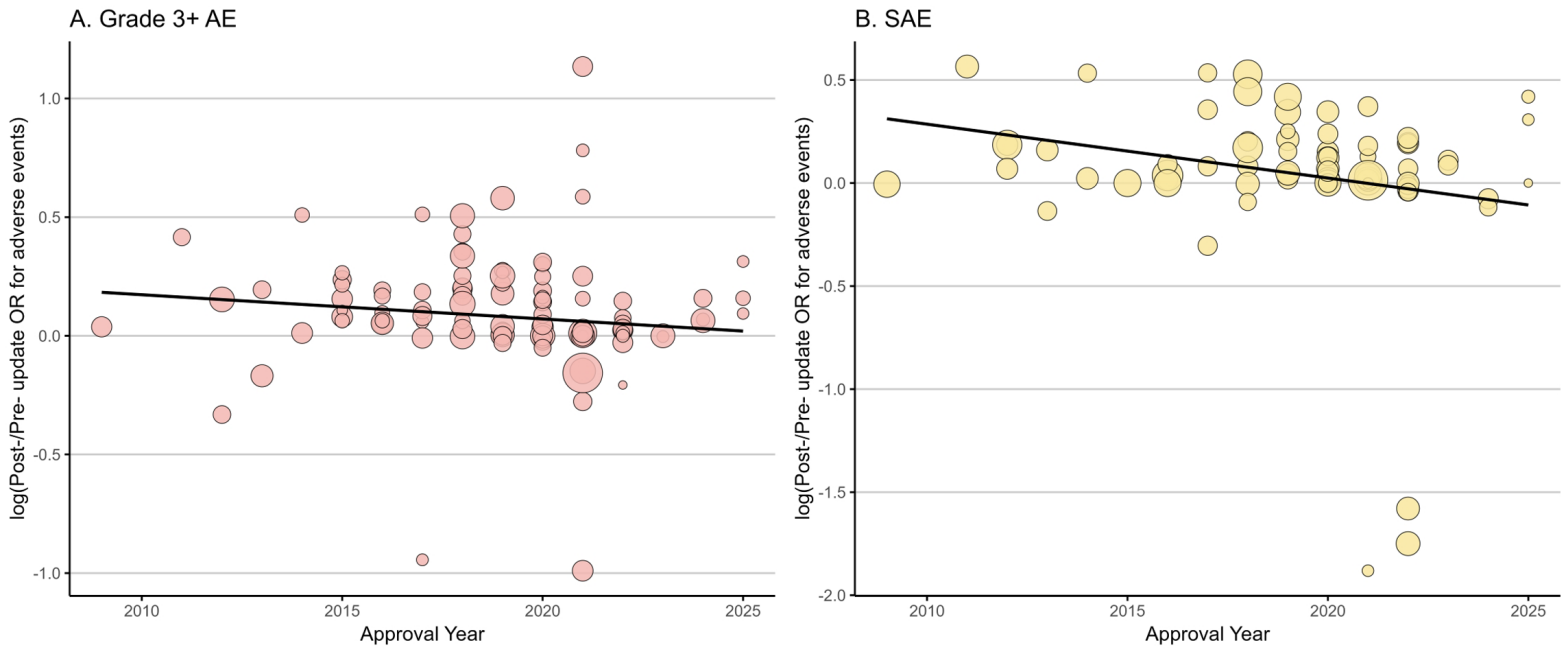
Meta-regression of changes in relative treatment effects Between updated and initial analyses by year of FDA approval **(A)** Grade 3 + AEs; **(B)** SAEs. Each circle represents one pairwise comparison, with circle size proportional to its inverse-variance weight in the random-effects meta-regression. The solid line shows the fitted regression of log(ROR) on approval year. AE, adverse event; ROR, ratio of odds ratios (updated OR divided by initial OR); SAE, serious adverse event.

As shown in Figure 5, RORs decreased with later approval years for both SAEs (log(ROR) = 52.821–0.0267 × Year; *β* = −0.027, *p* = 0.009) and grade ≥3 AEs (log(ROR) = 20.69–0.0102 × Year; *β* = −0.01, *p* = 0.23), suggesting improved relative tolerability of newer treatments. For other covariates, univariable meta-regression results for grade ≥3 AEs and SAE are summarized in Supplementary Tables S10, S11, respectively. Multivariable meta-regression findings are summarized in Supplementary Tables S12, S13, for grade ≥3 AEs, both crossover allowance (*β* = 0.108, *p* = 0.041) and AE type (*β* = 0.099, *p* = 0.066) were associated with higher RORs, while other covariates showed no significant associations. These findings indicate that trials permitting patient crossover, or reporting all-cause rather than treatment-related AEs, tended to exhibit a greater increase in the relative toxicity of the experimental group compared with the control group after data updates. In contrast, studies restricting crossover or focusing on treatment-related AEs showed smaller or reduced shifts in relative AE risk following data maturation. For SAE, a longer initial follow-up time (*β* = −0.013, *p* = 0.002) and the metastatic setting (*β* = −0.298, *p* = 0.003) were associated with lower RORs, indicating that trials with more mature early follow-up or those enrolling metastatic populations tended to show a reduction in the relative SAE risk after data updates. In contrast, larger sample size (*β* = 0.176, *p* = 0.026), longer follow-up duration (*β* = 0.006, *p* = 0.018), all-cause AE type (*β* = 0.220, *p* = 0.010), and blinded design (*β* = 0.217, *p* = 0.007) were associated with higher RORs, suggesting that in larger, longer, and blinded studies—especially those reporting all-cause AEs—the relative toxicity of the experimental group compared with the control group became more pronounced after the data were updated.

The TDP equation (full model in Supplementary Result S1) shows that pre-approval design features dominate predicted drift: open-label design (+0.22), all-cause AE reporting (+0.22), large sample size (+0.18), and first-line setting (+0.16) each independently raise log-ROR, whereas metastatic disease (−0.30) and longer initial follow-up (−0.013/month) attenuate it. Updated follow-up horizon contributes modestly (+0.006/month). SAEs was the only endpoint showing mild-to-moderate heterogeneity, for which meta-regression was performed to identify potential sources of variability. The analysis explained approximately 33% of the between-study variance, reducing *I*^2^ from 47.2 to 36.5%.

## Discussion

In this systematic retrospective analysis of 99 pivotal RCTs contributing 106 pairwise comparisons, we characterized how the relative safety profile of FDA-approved oncology therapeutics evolved between the data cut that supported marketing authorization and the most recent post-approval update of the same study. The odds of grade ≥3 AEs in experimental arms relative to controls rose from 1.09 (95% CI, 0.93–1.22) at approval to 1.29 (1.03–1.43) with extended follow-up, yielding a within-trial meta-analytic ROR of 1.095 (1.038–1.156). A directionally concordant pattern was observed for SAEs, with pooled ORs shifting from 1.32 (1.21–1.42) to 1.45 (1.27–1.59) and an ROR of 1.06 (0.97–1.17). In 78 of 103 grade ≥3 comparisons and 49 of 70 SAE comparisons, the experimental arm accumulated a disproportionately greater burden of severe toxicity as trials matured. These findings indicate that the comparative toxicity profile available at approval systematically underrepresents the harm ultimately experienced by patients once trials reach greater data maturity (28, 29). Operationalised as the TDP, our model offers a transparent, pre-approval estimate of expected post-marketing toxicity drift, supporting risk-based prioritisation of confirmatory follow-up.

A notable granular finding is the divergence between all-cause and treatment-related AEs. RORs were substantially larger for all-cause events (grade ≥3: 1.16; SAEs: 1.22) than for events adjudicated as treatment-related (grade ≥3: 1.05; SAEs: 0.94). Two non-mutually-exclusive explanations warrant consideration. First, prolonged on-treatment time in the experimental arm inevitably expands the window during which any clinical event—intercurrent illness, infection, progression-related complications—can be recorded, even if unrelated to the drug itself. Second, causality adjudication is investigator-dependent and susceptible to attribution drift as trials mature, with late-emerging or mechanistically unexpected toxicities disproportionately coded as “unrelated” (30). Because patients, clinicians, and payers experience all-cause morbidity regardless of attribution, the all-cause signal may represent the more clinically faithful estimate of the incremental harm associated with newer regimens (30).

The subgroup patterns are biologically coherent. Increased RORs clustered in crossover-permissive designs, blinded trials, medium-sized studies, subsequent-line settings, and in lung, prostate, and breast cancer indications. Crossover, by shortening control-arm exposure once progression occurs, truncates opportunities to capture late toxicity on the comparator while leaving experimental-arm event accrual unaffected—mechanically widening the toxicity gap with time (31). Blinded designs, which more commonly enroll relatively healthier populations under placebo or supportive-care comparators, further magnify contrasts in delayed AE accrual (31). Larger and longer trials simply generate more person-time for rare or cumulative events to surface, consistent with the statistical reality that low-incidence toxicities scale with cumulative exposure (32). In prostate and breast cancer, where indolent disease biology permits years of continuous therapy with androgen-receptor pathway inhibitors, CDK4/6 inhibitors, PARP inhibitors, and endocrine–targeted combinations, the extended exposure windows are particularly prone to revealing cumulative hematologic, cardiovascular, and endocrine toxicities that are absent from short-follow-up datasets (33).

Our meta-regression identified a decreasing trend in SAE RORs with later approval years (*β* = −0.027, *p* = 0.009). Several forces likely contribute. Agents approved in more recent cycles increasingly comprise molecularly precise targeted therapies, antibody–drug conjugates with refined linker–payload chemistry, and checkpoint inhibitors deployed with maturing toxicity-management paradigms, each associated with narrower relative toxicity differentials against contemporary controls (34). Regulatory and sponsor learning has also advanced: protocolized irAE management, mandatory premedication and prophylaxis strategies for ADCs, and risk-based monitoring frameworks have measurably curtailed severe-event rates (35, 36). Control-arm composition has simultaneously shifted toward more intensive regimens (e.g., chemoimmunotherapy, doublet or triplet combinations), narrowing the toxicity gradient against which experimental arms are benchmarked (34). The absence of a similar temporal decline for grade ≥3 AEs may reflect the fact that lower-threshold events are dominated by on-target, mechanism-based toxicities that scale with cumulative exposure regardless of generational refinements (32).

The excess severe toxicity that surfaces in experimental arms with longer follow-up is mechanistically intelligible. Antibody–drug conjugates produce delayed interstitial lung disease, cytopenias, and ocular surface toxicity whose incidence rises with prolonged exposure, as demonstrated across the trastuzumab deruxtecan and enfortumab vedotin programs (35, 37, 38). Immune checkpoint inhibitors generate a distinctive profile of chronic immune-related events—thyroid dysfunction, hypophysitis, inflammatory arthritis, and insulin-dependent diabetes—that often persist or first appear months to years after treatment discontinuation; in long-term follow-up of adjuvant anti–PD-1 melanoma cohorts, a substantial proportion of irAEs remained active years after therapy ended (9, 10). Bispecific T-cell engagers and CAR-T constructs introduce protracted cytopenias, hypogammaglobulinemia, and opportunistic infections that only become evident with extended immune-reconstitution windows (39, 40). Multitargeted kinase inhibitors exert sustained off-target pressure on cardiovascular, dermatologic, and endocrine tissues, producing cumulative hypertension, thromboembolism, and thyroid dysfunction whose incidence rises with treatment duration (41). Combination regimens compound these class-specific liabilities through overlapping or synergistic toxicity mechanisms that are unpredictable from monotherapy datasets (42). Control arms—typically short-course chemotherapy, endocrine monotherapy, placebo, or supportive care—generate acute, largely reversible toxicities whose rates plateau once active treatment ends, creating an inherent asymmetry in how experimental and control arms accumulate AEs over time.

The meta-regression observation that larger sample size and longer total follow-up were associated with higher SAE RORs, whereas greater initial follow-up maturity and enrollment of metastatic populations were associated with lower RORs, is internally consistent. Early safety databases constrained to small samples and short exposure lack the statistical and temporal resolution to capture low-frequency or latent events, so subsequent data cuts expose toxicities that were structurally invisible at approval (32). Conversely, when the initial analysis itself reflects relatively mature exposure, the scope for further AE accrual is mechanically bounded. In metastatic cohorts, competing mortality from disease progression shortens time on therapy—particularly in control arms receiving palliative regimens—thereby attenuating the arithmetic expansion of the experimental-versus-control toxicity contrast otherwise observed with extended follow-up. These patterns reinforce the interpretation that the magnitude of the AE signal detected at approval is not a stable property of the regimen but a function of the evidentiary window through which it is observed.

A seeming paradox warrants direct comment: blinding, all-cause AE ascertainment, larger enrollment, longer follow-up, and non-metastatic settings predicted greater, not smaller, post-approval drift. The resolution lies in distinguishing the statistical precision of an interim estimate from the temporal completeness of its underlying event history—rigorous designs sharpen estimates of events already observed but cannot register events that have not yet occurred. Late-emerging immune-related endocrinopathies, antibody drug conjugate-associated interstitial lung disease, kinase-inhibitor cardiovascular toxicity, and protracted cytopenias following chimeric antigen receptor T-cell therapy are structurally absent from short-exposure datasets regardless of design quality, and extended follow-up populates this missing tail rather than correcting a noisy estimate. Three mechanics translate this principle into the directions observed. First, blinding and all-cause ascertainment jointly suppress attribution bias along complementary axes—blinding neutralizes the observer, who in open-label trials disproportionately reclassifies unexpected late events as unrelated, while all-cause counting bypasses investigator-assigned causality altogether; both preserve the late-event signal that treatment-related coding erodes, accounting for the wider all-cause versus treatment-related AE divergence noted above. Second, larger and longer trials accrue the person-time required for low-incidence, latency-driven toxicities to surface, providing the long-term safety infrastructure to detect events invisible at pivotal interim cuts. Third, crossover asymmetrically truncates control-arm exposure, inflating the OR with time independently of design rigor, with the metastatic-setting attenuation as its symmetric counterpart in which competing mortality compresses on-treatment time and removes the substrate for late-event accrual. Read this way, drift is not instability of high-quality trials but the faithful disclosure, through superior detection infrastructure, of toxicity that was always present yet temporally invisible at approval—smaller, shorter, open-label trials appear more stable only because they are structurally blind to the events that better-resourced trials are powered to reveal.

Collectively, these findings carry direct regulatory implications. Expedited pathways rest on the premise that early efficacy signals justify accelerated patient access despite an inherently immature safety dataset, with the understanding that post-marketing evidence will refine the benefit–risk portrait (28, 43). Our results provide quantitative support for the concern that the relative safety profile presented at approval is not simply noisier than its mature counterpart but systematically biased toward underestimation of comparative harm. Post-approval labeling analyses have shown that safety-related label changes are frequent and often reflect toxicities not fully characterized at launch, reinforcing the need for structured evidence-renewal mechanisms (43). Predefined safety-update milestones tied to cumulative exposure rather than to calendar time, mandatory reporting of delayed and immune-related toxicity cohorts, and routine integration of real-world pharmacovigilance into confirmatory assessments would better align regulatory practice with an evidence-maturity principle. For practicing clinicians, the findings argue for protocolized surveillance of cumulative and delayed toxicities—particularly endocrine, cardiovascular, pulmonary, and immune-mediated events—well beyond the exposure durations represented in pivotal-trial publications, and for transparent communication that toxicity rates available at approval should be regarded as floor, not ceiling, estimates of long-term harm (10, 44).

### Limitations

First, by requiring at least one published updated dataset, our sample may be subject to publication bias, as trials with unremarkable post-approval safety profiles may be less likely to be reported in full; the true increase in the relative toxicity of novel agents may therefore be larger than observed. Second, reliance on aggregate event counts rather than individual patient data precluded analysis of time-to-first-event, recurrent events, and AE resolution dynamics. Third, because the sample was restricted to US FDA approvals, generalizability to other regulators requires separate validation, given jurisdictional differences in evidentiary standards and expedited-pathway design. Finally, the ROR framework captures within-trial relative change but cannot disentangle true biological toxicity accumulation from the mechanical effect of longer exposure windows on event capture; exposure-adjusted analyses of individual-patient data will be needed to resolve this question.

## Conclusion

To our knowledge, this is the first systematic, within-trial quantification showing that the relative toxicity of FDA-approved oncology therapeutics versus their comparators intensifies from the approval data cut to later post-approval updates. Across drug classes, tumor types, and approval cohorts, experimental arms accumulated disproportionately greater grade ≥3 and serious AEs as trials matured, with the signal most pronounced for all-cause events, in crossover-permissive and blinded designs, in prostate, breast, and lung cancer, and in larger, longer-follow-up studies. Although differentials have narrowed for more recently approved agents, the comparative safety information available at approval systematically underrepresents the harm ultimately experienced by patients. These findings support a shift from static approval to evidence-maturity–based dynamic regulation, with predefined safety-update milestones, intensified post-marketing surveillance of delayed toxicities, and routine integration of mature trial and real-world data into life-cycle benefit–risk assessment.

## Data Availability

The original contributions presented in the study are included in the article/Supplementary material, further inquiries can be directed to the corresponding author.

## Footnotes

1
https://doi.org/10.17605/OSF.IO/3YQGC
