# Supplementary material for: Longitudinal changes in the relative toxicity of FDA-approved oncology therapeutics: evidence from paired initial and updated RCT reports
Source: Front Public Health. 2026 Jul 6;14:1882548. doi: 10.3389/fpubh.2026.1882548 (PMC13381507; doi:10.3389/fpubh.2026.1882548)
Supplement: Supplementary file 1 [file Table_1.DOCX]

**Supplementary Material**

[Table S1. Prespecified variables in meta-regression analyses of AE 2](#_Toc14468)

[Table S2 Prespecified subgroups and rationale for stratification of AE analyses 4](#_Toc16393)

[Table S3 Characteristics of 99 clinical trials included in the analysis 6](#_Toc5728)

[Table S4 Characteristics of 99 clinical trials included in the analysis 15](#_Toc9748)

[Table S5 Summary of the systematic search strategy 18](#_Toc29309)

[Table S6 Overall and Subgroup meta-analysis of grade 3+ adverse-event odds ratios (OR) before and after data updates 20](#_Toc16292)

[Table S7 Overall and Subgroup Meta-analysis of serious adverse-event odds ratios (OR) before and after data updates 22](#_Toc4664)

[Table S8 Overall and Subgroup Meta-analysis of ROR (Ratio of updated OR to initial OR) for grade 3+ AE 24](#_Toc14547)

[Table S9 Overall and Subgroup Meta-analysis of ROR (Ratio of updated OR to initial OR) for serious AE 26](#_Toc17581)

[Table S10 Univariable meta-regression results of Grade 3+ AE by individual covariates 28](#_Toc21006)

[Table S11 Univariable meta-regression results of SAE by individual covariates 28](#_Toc18257)

[Table S12 Multivariable meta-regression results of grade 3+AE 29](#_Toc16071)

[Table S13 Multivariable meta-regression results of SAE 30](#_Toc1108)

[Method S1 Toxicity Drift Predictor, Full Methods 31](#_Toc23252)

[Result S1. Variable coding and meta-regression coefficients 32](#_Toc8465)

# Table S1. Prespecified variables in meta-regression analyses of AE

| Endpoint | Variable | Variable type | Definition / Coding | Rationale or interpretation |
| --- | --- | --- | --- | --- |
| AE (Grade ≥3 or Serious) | FDA approval year | Continuous (numeric) | Calendar year of FDA approval for the investigational regimen | Represents therapeutic‑era or time‑trend effect |
| AE (Grade ≥3 or Serious) | Five‑year disease survival rate | Continuous (proportion 0–1) | Background long‑term survival rate of the underlying cancer type | The 5-year survival rates by indication were obtained from the SEER database (https://seer.cancer.gov/) |
| AE (Grade ≥3 or Serious) | Trial phase | Categorical (phase II vs phase III) | phase II = early‑stage exploratory; phase III = confirmatory | Proxy for trial rigor and maturity of evidence |
| AE (Grade ≥3 or Serious) | Treatment line | Categorical (binary) | First‑line = 0; Later‑line = 1 (subsequent or relapsed setting) | Captures previous therapy exposure and clinical context |
| AE (Grade ≥3 or Serious) | Metastatic setting | Categorical (binary) | Non‑metastatic = 0; Metastatic = 1 | Represents baseline disease extent and prognosis |
| AE (Grade ≥3 or Serious) | Blinding status | Categorical (binary) | Open‑label = 0; Blinded = 1 | Indicator of potential performance / assessment bias |
| AE (Grade ≥3 or Serious) | Crossover allowance | Categorical (binary) | No = 0; Yes = 1 | Tests whether crossover diluted differences |
| AE (Grade ≥3 or Serious) | Follow‑up duration | Continuous (months) | Difference between post‑ and pre‑cutoff times | Reflects observation time and data maturity |
| AE (Grade ≥3 or Serious) | AE type | Categorical (binary) | Treatment‑related = 0; All‑cause = 1 | Differentiates causality scope of AE outcomes |
| AE (Grade ≥3 or Serious) | Trial sample size | Categorical (ordinal) | Small (< 100)=1, Medium (100–600)=2, Large (> 600)=3 | Represents study size and statistical power of individual trials |
| AE (Grade ≥3 or Serious) | Follow-up time | Continuous (months) | Median follow-up time of RCT, including both the initial and updated analyses | Represents overall time duration of RCT |

Note, OS = overall survival; PFS = progression-free survival (including EFS); AE = adverse events (including Grade ≥3 and serious AE analyses)

# Table S2 Prespecified subgroups and rationale for stratification of AE analyses

| Endpoint | Subgroup variable | Levels / Coding rule | Rationale / Reference |
| --- | --- | --- | --- |
| SAE / Grade ≥3 AE | FDA approval year | ≤ 2015 (Pre‑Immunotherapy era); 2016–2020 (Immune‑oncology expansion); ≥ 2021 (Modern combination and biomarker‑driven era) | Reflects chronological evolution of oncology therapeutics and regulatory landscapes |
| SAE / Grade ≥3 AE | 5‑year survival rate | Low (< 30%), Intermediate (30–69%), High (≥ 70%) | The 5-year survival rates by indication were obtained from the SEER database (https://seer.cancer.gov/), with classification criteria based on Siegel et al^2^ |
| SAE / Grade ≥3 AE | Trial sample size | Small (< 100), Medium (100–600), Large (> 600) | Represents trial scale, event maturity, and statistical precision^3^ |
| SAE / Grade ≥3 AE | Treatment line | First‑line vs later‑line | Reflects line of therapy and prior treatment exposure |
| SAE / Grade ≥3 AE | Treatment category | Defined by mechanism of experimental and control regimens (e.g., IO vs chemotherapy vs targeted therapy); regimens with < 4 trials pooled as “Others” | Captures mechanistic class and control type differences, not specific drug names |
| SAE / Grade ≥3 AE | Trial phase | phase II vs phase III | Distinguishes trial design rigor and maturity of clinical evidence |
| SAE / Grade ≥3 AE | Blinding | Blinded vs Open‑label | Evaluates risk of performance and detection bias |
| SAE / Grade ≥3 AE | Crossover allowance | Yes vs No | Tests whether patient crossover attenuates between‑group effects |
| SAE / Grade ≥3 AE | Disease type | Rare (< 5 studies) merged as “Others” | Reduces heterogeneity from small tumor groups |
| SAE / Grade ≥3 AE | Metastatic status | Metastatic vs Non‑metastatic | Accounts for disease stage and baseline prognosis |
| SAE / Grade ≥3 AE | Follow‑up duration | Short (< 12 mo), Medium (12–24 mo), Long (> 24 mo) | Reflects timing and completeness of endpoint assessment |
| AE (Serious or Grade ≥3) | AE type | 0 = Treatment‑related; 1 = All‑cause | Distinguishes causal scope of AE reporting |

Note, OS = overall survival; PFS = progression-free survival (including EFS); AE = adverse events (including Grade ≥3 and serious AE analyses)

# Table S3 Characteristics of 99 clinical trials included in the analysis

| No of trial | Trial | NCT | Blinding, phase | FDA Approve Year | Indication | Treatment Regimen | Control Regimen | Treatment Group Sample Size | Control Regimen Sample Size |
| --- | --- | --- | --- | --- | --- | --- | --- | --- | --- |
| 1 | ADAURA | NCT02511106 | double-blind, phase 3 | 12/18/2020 | EGFR-mutated (Ex19del or L858R) non–small-cell lung cancer | Osimertinib | Placebo | 339 | 343 |
| 2 | AG120-C-005 | NCT02989857 | double-blind, phase 3 | 8/25/2021 | IDH1‑mutant cholangiocarcinoma | Ivosidenib | placebo | 124 | 61 |
| 3 | ALEX | NCT02075840 | open-label, phase 3 | 11/6/2017 | ALK-positive non–small-cell lung cancer | Alectinib | Crizotinib | 152 | 151 |
| 4 | ALTA-1L | NCT02737501 | open-label, phase 3 | 5/22/2020 | ALK-positive non–small-cell lung cancer | Brigatinib | Crizotinib | 137 | 138 |
| 5 | ARCHES | NCT02677896 | double-blind, phase 3 | 12/16/2019 | Metastatic hormone-sensitive prostate cancer | Enzalutamide+androgen deprivation therapy | androgen deprivation therapy | 574 | 576 |
| 6 | ASCENT | NCT02574455 | open-label, phase 3 | 4/7/2021 | Relapsed or refractory metastatic triple-negative breast cancer | Sacituzumab govitecan | Single-agent chemotherapy of physician's choice | 235 | 233 |
| 7 | ATTRACTION-3 | NCT02569242 | open-label, phase 3 | 5/27/2022 | Unresectable advanced or recurrent esophageal squamous cell carcinoma | Nivolumab | Investigator's choice single-agent chemotherapy | 210 | 209 |
| 8 | BEACONCRC (DOU-CON) | NCT02928224 | open-label, phase 3 | 4/8/2020 | Metastatic colorectal cancer with BRAF V600E mutation | encorafenib + binimetinib + cetuximab | cetuximab + irinotecan or cetuximab + FOLFIRI | 224 | 221 |
| 8 | BEACONCRC (TRI-CON) | NCT02928224 | open-label, phase 3 | 4/8/2020 | Metastatic colorectal cancer with BRAF V600E mutation | encorafenib + cetuximab | cetuximab + irinotecan or cetuximab + FOLFIRI | 220 | 221 |
| 9 | BOLERO-2 | NCT00863655 | double-blind, phase 3 | 7/24/2012 | hormone receptor–positive, HER2‑negative advanced/metastatic breast cancer | Everolimus plus exemestane | Exemestane | 485 | 239 |
| 10 | BREAKWATER | NCT04607421 | open-label, phase 3 | 12/20/2024 | metastatic colorectal cancer with BRAF V600E mutation (stage IV) | Encorafenib+mFOLFOX6 | Standard of Care | 236 | 243 |
| 11 | CABOSUN | NCT01835158 | open-label, phase 2 | 12/19/2017 | Metastatic clear cell renal cell carcinoma | Cabozantinib | Sunitinib | 79 | 78 |
| 12 | CANDOR | NCT03158688 | open-label, phase 3 | 8/20/2020 | Relapsed or refractory multiple myeloma | Carfilzomib+dexamethasone+daratumumab | Carfilzomib + dexamethasone | 312 | 154 |
| 13 | CASPIAN | NCT03043872 | open-label, phase 3 | 3/27/2020 | Extensive-stage small-cell lung cancer | Durvalumab+platinum–etoposide | Platinum–etoposide | 268 | 269 |
| 14 | CheckMate 816 | NCT02998528 | open-label, phase 3 | 3/4/2022 | Resectable Non-Small Cell Lung Cancer | Nivolumab+platinum-doublet chemotherapy | Platinum-doublet chemotherapy | 179 | 179 |
| 15 | CheckMate 9LA | NCT03215706 | open-label, phase 3 | 5/26/2020 | advanced (stage IV or recurrent) non–small cell lung cancer | Nivolumab+ipilimumab+chemotherapy | chemotherapy | 361 | 358 |
| 16 | CheckMate025 | NCT01668784 | open-label, phase 3 | 2015 | Advanced or metastatic clear-cell renal-cell carcinoma | Nivolumab | Everolimus | 410 | 411 |
| 17 | CheckMate141 | NCT02105636 | open-label, phase 3 | 2016 | refractory squamous-cell carcinoma of the head and neck | Nivolumab | systemic therapy | 240 | 121 |
| 18 | CheckMate214 | NCT02231749 | open-label, phase 3 | 4/16/2018 | Previously untreated advanced (metastatic) clear-cell renal-cell carcinoma | Nivolumab | Sunitinib | 550 | 546 |
| 19 | CheckMate227 | NCT02477826 | open-label, phase 3 | 5/15/2020 | advanced non–small-cell lung cancer | Nivolumab+ipilimumab | Platinum‑doublet chemotherapy | 396 | 397 |
| 20 | CheckMate238 | NCT02388906 | double-blind, phase 3 | 12/20/2017 | stage IV melanoma | Nivolumab | Ipilimumab | 453 | 453 |
| 21 | CHECKMATE-274 | NCT02632409 | double-blind, phase 3 | 8/19/2021 | High‑risk muscle‑invasive urothelial carcinoma | Nivolumab | Placebo | 353 | 356 |
| 22 | CHECKMATE-648 (NC-C) | NCT03143153 | open-label, phase 3 | 5/31/2022 | metastatic esophageal squamous-cell carcinoma | Nivolumab+Chemotherapy | Chemotherapy | 321 | 324 |
| 22 | CHECKMATE-648 (NI-C) | NCT03143153 | open-label, phase 3 | 5/31/2022 | metastatic esophageal squamous-cell carcinoma | Nivolumab+Ipilimumab | Chemotherapy | 325 | 324 |
| 23 | CheckMate649 | NCT02872116 | open-label, phase 3 | 4/16/2021 | HER2‑negative gastric, gastro‑oesophageal junction, or oesophageal adenocarcinoma | Nivolumab+chemotherapy | Chemotherapy | 789 | 792 |
| 24 | CheckMate743 | NCT02899299 | open-label, phase 3 | 10/2/2020 | malignant pleural mesothelioma | Nivolumab+ipilimumab | Platinum+pemetrexed | 303 | 302 |
| 25 | CheckMate9ER | NCT03141177 | open-label, phase 3 | 1/22/2021 | advanced renal-cell carcinoma with a clear-cell component | Nivolumab+cabozantinib | Sunitinib | 323 | 328 |
| 26 | CLL | NCT02242942 | open-label, phase 3 | 5/15/2019 | untreated chronic lymphocytic leukemia | Venetoclax+obinutuzumab | Chlorambucil–Obinutuzumab | 216 | 216 |
| 27 | coBRIM | NCT01689519 | double-blind，phase phase 3 | 2015 | melanoma | Vemurafenib + cobimetinib | Vemurafenib + placebo | 247 | 248 |
| 28 | CodeBreaK 300 (240-C) | NCT05198934 | open-label, phase 3 | 1/16/2025 | metastatic colorectal cancer with KRAS G12C mutation | 240-mg Sotorasib–Panitumumab | Standard Care | 53 | 54 |
| 28 | CodeBreaK 300 (960-C) | NCT05198934 | open-label, phase 3 | 1/16/2025 | metastatic colorectal cancer with KRAS G12C mutation | 960-mg Sotorasib–Panitumumab | Standard Care | 53 | 54 |
| 29 | COLUMBUS (ENB-EN) | NCT01909453 | open-label, phase 3 | 6/27/2018 | metastatic cutaneous melanoma with BRAFV600E or BRAFV600K mutation | Encorafenib 450 mg+binimetinib 45 mg | Encorafenib | 192 | 194 |
| 29 | COLUMBUS (ENB-VEM) | NCT01909453 | open-label, phase 3 | 6/27/2018 | metastatic cutaneous melanoma with BRAFV600E or BRAFV600K mutation | Encorafenib 450 mg+binimetinib 45 mg | Vemurafenib | 192 | 191 |
| 30 | COMBI-d | NCT01584648 | double-blind, phase 3 | 6/22/2022 | Unresectable stage IIIC or stage IV BRAF V600E or V600K-mutant metastatic melanoma | Dabrafenib+Trametinib | Dabrafenib | 211 | 212 |
| 31 | COSMIC-311 | NCT03690388 | double-blind，phase phase 3 | 9/17/2021 | radioiodine-refractory differentiated thyroid cancer | Cabozantinib | Placebo | 125 | 62 |
| 32 | COU-AA-302 | NCT00887198 | double-blind, phase 3 | 12/10/2012 | Metastatic castration-resistant prostate cancer | Abiraterone acetate+Prednisone | Prednisone | 546 | 542 |
| 33 | CROWN | NCT03052608 | open-label，phase phase 3 | 3/3/2021 | ALK-Positive Non–Small-Cell Lung Cancer | Lorlatinib | Crizotinib | 149 | 147 |
| 34 | CRYSTAL | NCT00154102 | open-label, phase 3 | 10/15/2021 | metastatic colorectal cancer | Cetuximab+FOLFIRI | FOLFIRI | 599 | 599 |
| 35 | DESTINY-Breast03 | NCT03529110 | open-label，phase phase 3 | 5/4/2022 | HER2-positive metastatic breast cancer | Trastuzumab deruxtecan | Trastuzumab emtansine | 261 | 263 |
| 36 | EMBRACA | NCT01945775 | open-label, phase 3 | 12/14/2018 | metastatic breast cancer with a germline BRCA1/2 mutation | Acalabrutinib monotherapy | Obinutuzumab chlorambucil | 179 | 177 |
| 37 | EMILIA | NCT00829166 | open-label, phase 3 | 2/22/2013 | metastatic HER2-positive breast cancer | Trastuzumab emtansine | Lapatinib+Capecitabine | 495 | 496 |
| 38 | EMPOWER-Lung1 | NCT03088540 | open-label，phase 3 | 2/22/2021 | aNSCLC | Cemiplimab | Chemotherapy | 283 | 280 |
| 39 | EMPOWER-Lung3 | NCT03409614 | double-blind，phase 3 | 11/8/2022 | Advanced NSCLC | Cemiplimab+Chemotherapy | Chemotherapy | 312 | 154 |
| 40 | FLAURA | NCT02296125 | double-blind，phase phase 3 | 4/18/2018 | EGFR mutation–positive advanced non–small-cell lung cancer | Osimertinib | Standard EGFR-TKI | 279 | 277 |
| 41 | GADOLIN | NCT01059630 | open-label，phase phase 3 | 3/1/2016 | Rituximab-refractory indolent non-Hodgkin lymphoma | Obinutuzumab plus bendamustine | Bendamustine | 194 | 202 |
| 42 | GALLIUM | NCT01332968 | open-label, phase 3 | 12/17/2021 | untreated, advanced-stage CD20-positive follicular lymphoma | obinutuzumab+chemotherapy | Rituximab+chemotherapy | 601 | 601 |
| 43 | HIMALAYA (DUR-SOR) | NCT03298451 | open-label, phase 3 | 10/24/2022 | Unresectable hepatocellular carcinoma | Durvalumab | Sorafenib | 389 | 389 |
| 43 | HIMALAYA (STR-SOR) | NCT03298451 | open-label, phase 3 | 10/24/2022 | Unresectable hepatocellular carcinoma | Tremelimumab+Durvalumab | Sorafenib | 393 | 389 |
| 44 | ICARIA-MM | NCT02990338 | open-label，phase phase 3 | 3/2/2020 | Relapsed and refractory multiple myeloma | Isatuximab，pomalidomide and dexamethasone | Pomalidomide plus dexamethasone | 154 | 153 |
| 45 | IKEMA | NCT03275285 | open-label，phase phase 3 | 3/31/2021 | Relapsed multiple myeloma | Isatuximab, pomalidomide and dexamethasone | Carfilzomib–dexamethasone | 179 | 123 |
| 46 | IMbrave150 | NCT03434379 | open-label，phase phase 3 | 5/29/2020 | Unresectable hepatocellular carcinoma | Atezolizumab and bevacizumab | Sorafenib | 336 | 165 |
| 47 | IMCgp100-202 | NCT03070392 | open-label，phase phase 3 | 1/25/2022 | Metastatic Uveal Melanoma | Tebentafusp | Pembrolizumab | 252 | 126 |
| 48 | IMpassion130 | NCT02425891 | double-blind，phase phase 3 | 3/8/2019 | Advanced Triple-Negative Breast Cancer | Atezolizumab plus nab-paclitaxel | Placebo plus nab-paclitaxel | 451 | 451 |
| 49 | IMpower010 | NCT02486718 | open-label，phase 3 | 10/15/2021 | Stage II-IIIA non-small-cell lung cancer | Atezolizumab | Best supportive care | 507 | 498 |
| 50 | IMpower110 | NCT02409342 | open-label，phase 3 | 5/18/2020 | non–small-cell lung cancer | Atezolizumab | Chemotherapy | 285 | 287 |
| 51 | IMpower133 | NCT02763579 | double-blind，phase 3 | 3/18/2019 | Extensive-Stage Small-Cell Lung Cancer | Atezolizumab plus carboplatin and etoposide | Carboplatin and etoposide | 201 | 202 |
| 52 | IMspire150 | NCT02908672 | double-blind，phase 3 | 7/30/2020 | Advanced BRAFV600 mutation positive melanoma | Atezolizumab, vemurafenib, and cobimetinib | Vemurafenib, and cobimetinib | 256 | 258 |
| 53 | INAVO120 | NCT04191499 | double-blind, phase 3 | 10/10/2024 | PIK3CA-mutated, hormone receptor–positive, HER2-negative metastatic breast cancer | Inavolisib+Palbociclib+Fulvestrant | Palbociclib+Fulvestrant | 161 | 164 |
| 54 | INO-VATEALL | NCT01564784 | open-label，phase 3 | 8/17/2017 | Acute Lymphoblastic Leukemia | Inotuzumab Ozogamicin | Sandard intensive chemotherapy | 164 | 162 |
| 55 | JAVELIN 101 | NCT02684006 | open-label, phase 3 | 5/14/2019 | Advanced renal-cell carcinoma | Avelumab plus axitinib | Sunitinib | 442 | 444 |
| 56 | JUPITER-02 | NCT03581786 | double-blind, phase 3 | 10/30/2023 | metastatic nonkeratinizing nasopharyngeal carcinoma | Toripalimab+Gemcitabine+Cisplatin | Gemcitabine+Cisplatin | 146 | 143 |
| 57 | KATHERINE | NCT01772472 | open-label, phase 3 | 5/6/2019 | HER2-positive early breast cancer | Trastuzumab emtansine | Trastuzumab | 743 | 743 |
| 58 | KEYNOTE-002 (PEM Q2-CON) | NCT01704287 | double-blind, phase 3 | 2015 | Melanoma | Pembrolizumab | Chemotherapy | 179 | 171 |
| 58 | KEYNOTE-002 (PEM Q3-CON) | NCT01704287 | double-blind，phase 3 | 2015 | Melanoma | Pembrolizumab | Chemotherapy | 178 | 171 |
| 59 | KEYNOTE-010 | NCT01905657 | open-label, phase 3 | 2015 | Lung Cancer | Pembrolizumab | Chemotherapy | 682 | 309 |
| 60 | KEYNOTE-024 | NCT02142738 | open-label, phase 3 | 4/11/2019 | Stage IV Non–Small-Cell Lung Cancer | Pembrolizumab | Chemotherapy | 154 | 151 |
| 61 | KEYNOTE-042 | NCT02220894 | open-label, phase 3 | 4/11/2019 | Advanced or metastatic non-small-cell lung cancer | Pembrolizumab | Chemotherapy | 637 | 637 |
| 62 | KEYNOTE-045 | NCT02256436 | open-label, phase 3 | 5/18/2017 | Advanced Urothelial Carcinoma | Pembrolizumab | Chemotherapy | 270 | 272 |
| 63 | KEYNOTE-048 (P-C) | NCT02358031 | open-label, phase 3 | 6/10/2019 | Recurrent or Metastatic Head and Neck Squamous Cell Carcinoma | Pembrolizumab monotherapy | Cetuximab-chemotherapy | 301 | 300 |
| 63 | KEYNOTE-048 (PC-C) | NCT02358031 | open-label, phase 3 | 6/10/2019 | Recurrent or Metastatic Head and Neck Squamous Cell Carcinoma | Pembrolizumab plus chemotherapy | Cetuximab plus chemotherapy | 281 | 300 |
| 64 | KEYNOTE-091 | NCT02504372 | double-blind，phase 3 | 2023 | Lung Cancer | Pembrolizumab | Supportive care/Placebo | 580 | 581 |
| 65 | KEYNOTE-189 | NCT02578680 | double-blind, phase 3 | 8/20/2018 | Metastatic Non–Small-Cell Lung Cancer | Pembrolizumab plus chemotherapy | Chemotherapy | 410 | 206 |
| 66 | KEYNOTE-355 | NCT02819518 | double-blind, phase 3 | 11/13/2020 | metastatic triple-negative breast cancer | Pembrolizumab | Placebo | 566 | 281 |
| 67 | KEYNOTE-407 | NCT02775435 | double-blind, phase 3 | 10/30/2018 | Squamous Non–Small-Cell Lung Cancer | Pembrolizumab plus chemotherapy | Chemotherapy | 278 | 281 |
| 68 | KEYNOTE-522 | NCT03036488 | double-blind, phase 3 | 7/26/2021 | Triple-Negative Breast Cancer | Pembrolizumab plus Chemotherapy | Chemotherapy | 784 | 390 |
| 69 | KEYNOTE-564 | NCT03142334 | double-blind, phase 3 | 11/17/2021 | Renal-Cell Carcinoma | Pembrolizumab | Placebo | 496 | 498 |
| 70 | KEYNOTE-590 | NCT03189719 | double-blind，phase 3 | 2021 | Gastric Cancer | Pembrolizumab-Chemotherapy | Chemotherapy | 370 | 370 |
| 71 | KEYNOTE-775 | NCT03517449 | open-label, phase 3 | 7/21/2021 | Advanced Endometrial Cancer | Lenvatinib plus Pembrolizumab | Chemotherapy | 411 | 416 |
| 72 | KEYNOTE-A18 | NCT04221945 | double-blind, phase 3 | 1/12/2024 | locally advanced cervical cancer | Pembrolizumab+chemoradiotherapy | Chemoradiotherapy | 528 | 530 |
| 73 | LATITUDE | NCT01715285 | double-blind, phase 3 | 2/7/2018 | Castration-Sensitive Prostate Cancer | Androgen-deprivation therapy plus Abiraterone | Androgen-deprivation therapy | 597 | 602 |
| 74 | MAIA | NCT02252172 | open-label, phase 3 | 6/27/2019 | Myeloma | Daratumumab plus lenalidomide and dexamethasone | Lenalidomide and dexamethasone alone | 368 | 369 |
| 75 | METEOR | NCT01865747 | open-label, phase 3 | 5/5/2016 | Advanced Renal-Cell Carcinoma | Cabozantinib | Everolimus | 330 | 328 |
| 76 | METRIC | NCT01245062 | open-label, phase 3 | 2013 | Unresectable stage IIIC or IV cutaneous melanoma with BRAF V600 | Trametinib | chemotherapy | 214 | 108 |
| 77 | monarchE | NCT03155997 | open-label, phase 3 | 10/12/2021 | HR+/HER2- early breast cancer | Abemaciclib plus endocrine therapy | Endocrine therapy | 2808 | 2829 |
| 78 | MURANO | NCT02005471 | open-label, phase 3 | 6/8/2018 | Chronic Lymphocytic Leukemia | Venetoclax plus rituximab | Bendamustine plus rituximab | 194 | 195 |
| 79 | OAK | NCT02008227 | open-label，phase 3 | 10/18/2016 | previously treated non-small-cell lung cancer | Atezolizumab | Docetaxel | 609 | 578 |
| 80 | OlympiAD | NCT02000622 | open-label，phase 3 | 1/12/2018 | metastatic breast cancer and a germline BRCA mutation | Olaparib | Chemotherapy | 205 | 97 |
| 81 | OPTiM | NCT00769704 | open-label, phase 3 | 10/27/2015 | Advanced Melanoma | Intralesional Talimogene laherparepvec | Granulocyte macrophage colony-stimulating factor | 295 | 141 |
| 82 | PACIFIC | NCT02125461 | double-blind，phase 3 | 2/16/2018 | stage III, unresectable non–small-cell lung cancer | Durvalumab | Placebo | 473 | 236 |
| 83 | PALOMA3 | NCT01942135 | double-blind, phase 3 | 3/31/2017 | HR-positive, HER2-negative early breast cancer | Palbociclib+Fulvestrant | Fulvestrant | 347 | 174 |
| 84 | PANORAMA | NCT01023308 | double-blind, phase 3 | 2015 | refractory multiple myeloma | Panobinostat+Bortezomib+Dexamethasone | Bortezomib+Dexamethasone | 387 | 381 |
| 85 | POLO | NCT02184195 | double-blind，phase 3 | 12/27/2019 | BRCA-Mutated Metastatic Pancreatic Cancer | Olaparib | Placebo | 92 | 62 |
| 86 | POPLAR | NCT01903993 | open-label，2 | 10/18/2016 | previously treated non-small-cell lung cancer | Atezolizumab | Docetaxel | 144 | 143 |
| 87 | PROFILE 1014 | NCT01526928 | open-label, phase 3 | 3/11/2016 | Previously untreated advanced non–small-cell lung cancer (NSCLC) with ALK | Crizotinib | Chemotherapy | 172 | 171 |
| 88 | PSMAfore | NCT04689828 | open-label, phase 3 | 3/28/2025 | PSMA-positive metastatic castration-resistant prostate cancer | [177Lu]Lu-PSMA-617 | abiraterone or enzalutamide | 234 | 234 |
| 89 | RELATIVITY-047 | NCT03470922 | double-blind, phase2-3 | 3/21/2022 | unresectable melanoma (unresectable stage III or stage IV) | Relatlimab+nivolumab | Nivolumab | 355 | 359 |
| 90 | SOPHIA | NCT02492711 | open-label，phase 3 | 12/16/2020 | ERRB2 (formerly HER2)–positive advanced breast cancer | Margetuximab | Trastuzumab | 266 | 270 |
| 91 | SPARTAN | NCT01946204 | double-blind，phase 3 | 2/14/2018 | metastatic, castration-sensitive prostate cancer | Apalutamide | Placebo | 806 | 401 |
| 92 | SSGXVIII | NCT00116935 | open-label, phase 3 | 1/31/2012 | KIT‑positive gastrointestinal stromal tumor | imatinib 36Months | imatinib 12Months | 198 | 199 |
| 93 | S-TRAC | NCT00375674 | double-blind, phase2-3 | 11/16/2017 | Locoregional clear-cell renal-cell carcinoma | Sunitinib | placebo | 309 | 306 |
| 94 | SUN 111 | NCT00428597 | double-blind, phase2-3 | 2011 | advanced pancreatic neuroendocrine tumors | Sunitinib | best supportive care | 86 | 85 |
| 95 | TITAN | NCT02489318 | double-blind，phase 3 | 9/17/2019 | Metastatic,Castration-Sensitive Prostate Cancer | Apalutamide to androgen-deprivation therapy | Androgen-deprivation therapy | 525 | 527 |
| 96 | TRANSFORM | NCT03575351 | open-label，phase 3 | 6/24/2022 | Relapsed or refractory large B-cell lymphoma | Lisocabtagene maraleucel | Immunochemotherapy | 92 | 92 |
| 97 | TROPiCS-02 | NCT03901339 | open-label，phase 3 | 2/3/2023 | HER2-Negative Metastatic Breast Cancer | Sacituzumab govitecan | chemotherapy | 272 | 271 |
| 98 | VISTA | NCT00111319 | open-label, phase 3 | 2014 | Newly diagnosed, symptomatic multiple myeloma | Bortezomib+melphalan+prednisone | Melphalan+prednisone | 344 | 338 |
| 99 | ZUMA-7 | NCT03391466 | open-label，phase 3 | 4/1/2022 | Large B-Cell Lymphoma | Axicabtagene ciloleucel | chemoimmunotherapy | 180 | 179 |

# Table S4 Characteristics of 99 clinical trials included in the analysis

| Trial | NCT | FDA Approve Year | Initial Publication Year | Updated Publication Year |
| --- | --- | --- | --- | --- |
| ADAURA | NCT02511106 | 12/18/2020 | 12/18/2020 | 12/18/2020 |
| AG120-C-005 | NCT02989857 | 8/25/2021 | 05/13/2020 | 09/23/2021 |
| ALEX | NCT02075840 | 11/6/2017 | 08/31/2017 | 05/11/2020 |
| ALTA-1L | NCT02737501 | 5/22/2020 | 09/25/2018 | 07/20/2020 |
| ARCHES | NCT02677896 | 12/16/2019 | 11/19/2019 | 03/07/2022 |
| ASCENT | NCT02574455 | 4/7/2021 | 04/22/2021 | 02/29/2024 |
| ATTRACTION-3 | NCT02569242 | 5/27/2022 | 10/30/2019 | 08/01/2022 |
| BEACONCRC (DOU-CON) | NCT02928224 | 4/8/2020 | 09/30/2019 | 10/08/2020 |
| BEACONCRC (TRI-CON) | NCT02928224 | 4/8/2020 | 09/30/2019 | 10/08/2020 |
| BOLERO-2 | NCT00863655 | 7/24/2012 | 18-May-13 | 9-Sep-14 |
| BREAKWATER | NCT04607421 | 12/20/2024 | 01/25/2025 | 06/25/2025 |
| CABOSUN | NCT01835158 | 12/19/2017 | 11/14/2016 | 02/23/2018 |
| CANDOR | NCT03158688 | 8/20/2020 | 18-Jul-20 | 19-Apr-23 |
| CASPIAN | NCT03043872 | 3/27/2020 | 10/04/2019 | 12/04/2020 |
| CheckMate 816 | NCT02998528 | 3/4/2022 | 05/26/2022 | 08/21/2025 |
| CheckMate 9LA | NCT03215706 | 5/26/2020 | 03/01/2021 | 08/11/2025 |
| CheckMate025 | NCT01668784 | 11/23/2015 | 09/25/2015 | 05/10/2020 |
| CheckMate141 | NCT02105636 | 11/10/2016 | 10/09/2016 | 04/10/2018 |
| CheckMate214 | NCT02231749 | 4/16/2018 | 03/21/2018 | 08/16/2019 |
| CheckMate227 | NCT02477826 | 5/15/2020 | 09/28/2019 | 01/23/2023 |
| CheckMate238 | NCT02388906 | 12/20/2017 | 30-Sep-17 | 19-Sep-20 |
| CHECKMATE-274 | NCT02632409 | 8/19/2021 | 27-Jul-21 | 11-Oct-24 |
| CHECKMATE-648 (NC-C) | NCT03143153 | 5/31/2022 | 21-Apr-24 | 12-Sep-25 |
| CHECKMATE-648 (NI-C) | NCT03143153 | 5/31/2022 | 21-Apr-24 | 12-Sep-25 |
| CheckMate649 | NCT02872116 | 4/16/2021 | 06/05/2021 | 03/23/2022 |
| CheckMate743 | NCT02899299 | 10/2/2020 | 01/21/2021 | 02/03/2022 |
| CheckMate9ER | NCT03141177 | 1/22/2021 | 03/04/2021 | 06/07/2022 |
| CLL | NCT02242942 | 5/15/2019 | 4-Jun-19 | 18-Apr-23 |
| coBRIM | NCT01689519 | 11/10/2015 | 11/13/2014 | 06/22/2021 |
| CodeBreaK 300 (240-C) | NCT05198934 | 1/16/2025 | 12/07/2023 | 04/11/2025 |
| CodeBreaK 300 (960-C) | NCT05198934 | 1/16/2025 | 12/07/2023 | 04/11/2025 |
| COLUMBUS (ENB-EN) | NCT01909453 | 6/27/2018 | 09/12/2018 | 07/21/2022 |
| COLUMBUS (ENB-VEM) | NCT01909453 | 6/27/2018 | 09/12/2018 | 07/21/2022 |
| COMBI-d | NCT01584648 | 6/22/2022 | 09/29/2014 | 05/05/2017 |
| COSMIC-311 | NCT03690388 | 9/17/2021 | 07/05/2021 | 08/30/2022 |
| COU-AA-302 | NCT00887198 | 12/10/2012 | 12/10/2012 | 01/16/2015 |
| CROWN | NCT03052608 | 3/3/2021 | 19-Nov-20 | 31-May-24 |
| CRYSTAL | NCT00154102 | 10/15/2021 | 04/02/2009 | 06/25/2014 |
| DESTINY-Breast03 | NCT03529110 | 5/4/2022 | 03/24/2022 | 04/26/2024 |
| EMBRACA | NCT01945775 | 12/14/2018 | 08/15/2018 | 06/10/2020 |
| EMILIA | NCT00829166 | 2/22/2013 | 11/08/2012 | 05/16/2017 |
| EMPOWER-Lung1 | NCT03088540 | 2/22/2021 | 02/13/2021 | 08/14/2023 |
| EMPOWER-Lung3 | NCT03409614 | 11/8/2022 | 03/05/2022 | 11/22/2022 |
| FLAURA | NCT02296125 | 4/18/2018 | 11/18/2017 | 11/21/2019 |
| GADOLIN | NCT01059630 | 3/1/2016 | 06/23/2016 | 03/27/2018 |
| GALLIUM | NCT01332968 | 12/17/2021 | 10/05/2017 | 05/19/2023 |
| HIMALAYA (DUR-SOR) | NCT03298451 | 10/24/2022 | 06/06/2022 | 02/19/2024 |
| HIMALAYA (STR-SOR) | NCT03298451 | 10/24/2022 | 06/06/2022 | 02/19/2024 |
| ICARIA-MM | NCT02990338 | 3/2/2020 | 11/14/2019 | 08/30/2022 |
| IKEMA | NCT03275285 | 3/31/2021 | 4-Jun-21 | 24-Jul-24 |
| IMbrave150 | NCT03434379 | 5/29/2020 | 05/14/2020 | 12/11/2021 |
| IMCgp100-202 | NCT03070392 | 1/25/2022 | 09/23/2021 | 10/29/2023 |
| IMpassion130 | NCT02425891 | 3/8/2019 | 11/15/2018 | 11/27/2019 |
| IMpower010 | NCT02486718 | 10/15/2021 | 09/20/2021 | 05/30/2025 |
| IMpower110 | NCT02409342 | 5/18/2020 | 10/01/2020 | 07/12/2021 |
| IMpower133 | NCT02763579 | 3/18/2019 | 09/25/2018 | 01/13/2021 |
| IMspire150 | NCT02908672 | 7/30/2020 | 06/13/2020 | 11/29/2022 |
| INAVO120 | NCT04191499 | 10/10/2024 | 10/31/2024 | 07/10/2025 |
| INO-VATEALL | NCT01564784 | 8/17/2017 | 06/13/2016 | 03/08/2019 |
| JAVELIN 101 | NCT02684006 | 5/14/2019 | 02/16/2019 | 04/06/2022 |
| JUPITER-02 | NCT03581786 | 10/30/2023 | 06/21/2021 | 09/18/2023 |
| KATHERINE | NCT01772472 | 5/6/2019 | 12/05/2018 | 01/16/2025 |
| KEYNOTE-002 (PEM Q2-CON) | NCT01704287 | 12/18/2015 | 24-Jun-15 | 18-Jul-17 |
| KEYNOTE-002 (PEM Q3-CON) | NCT01704287 | 12/18/2015 | 24-Jun-15 | 18-Jul-17 |
| KEYNOTE-010 | NCT01905657 | 10/02/2015 | 19-Dec-15 | 22-Feb-20 |
| KEYNOTE-024 | NCT02142738 | 4/11/2019 | 11/10/2016 | 04/19/2021 |
| KEYNOTE-042 | NCT02220894 | 4/11/2019 | 04/04/2019 | 10/28/2022 |
| KEYNOTE-045 | NCT02256436 | 5/18/2017 | 02/17/2017 | 12/06/2022 |
| KEYNOTE-048 (P-C) | NCT02358031 | 6/10/2019 | 10/11/2022 | 04/04/2025 |
| KEYNOTE-048 (PC-C) | NCT02358031 | 6/10/2019 | 10/11/2022 | 04/04/2025 |
| KEYNOTE-091 | NCT02504372 | 01/26/2023 | Oct-22 | Mar-22 |
| KEYNOTE-189 | NCT02578680 | 8/20/2018 | 04/16/2018 | 02/21/2023 |
| KEYNOTE-355 | NCT02819518 | 11/13/2020 | 21-Jul-22 | 5-Dec-20 |
| KEYNOTE-407 | NCT02775435 | 10/30/2018 | 09/25/2018 | 02/03/2023 |
| KEYNOTE-522 | NCT03036488 | 7/26/2021 | 02/10/2022 | 09/15/2024 |
| KEYNOTE-564 | NCT03142334 | 11/17/2021 | 08/19/2021 | 04/18/2024 |
| KEYNOTE-590 | NCT03189719 | 03/22/2021 | 28-Aug-21 | 21-Apr-25 |
| KEYNOTE-775 | NCT03517449 | 7/21/2021 | 01/19/2022 | 04/14/2023 |
| KEYNOTE-A18 | NCT04221945 | 1/12/2024 | 03/20/2024 | 09/14/2024 |
| LATITUDE | NCT01715285 | 2/7/2018 | 06/04/2017 | 04/12/2019 |
| MAIA | NCT02252172 | 6/27/2019 | 05/30/2019 | 10/13/2021 |
| METEOR | NCT01865747 | 5/5/2016 | 09/25/2015 | 06/05/2016 |
| METRIC | NCT01245062 | 05/29/2013 | 07/12/2012 | 12/05/2018 |
| monarchE | NCT03155997 | 10/12/2021 | 09/20/2020 | 12/06/2022 |
| MURANO | NCT02005471 | 6/8/2018 | 04/02/2018 | 09/29/2020 |
| OAK | NCT02008227 | 10/18/2016 | 12/12/2016 | 09/23/2020 |
| OlympiAD | NCT02000622 | 1/12/2018 | 06/04/2017 | 01/23/2019 |
| OPTiM | NCT00769704 | 10/27/2015 | 07/28/2017 | 01/15/2019 |
| PACIFIC | NCT02125461 | 2/16/2018 | 09/25/2018 | 02/02/2022 |
| PALOMA3 | NCT01942135 | 3/31/2017 | 7/2015 | 11/2018 |
| PANORAMA | NCT01023308 | 02/23/2015 | 09/19/2014 | 10/14/2016 |
| POLO | NCT02184195 | 12/27/2019 | 06/02/2019 | 07/14/2022 |
| POPLAR | NCT01903993 | 10/18/2016 | 03/09/2016 | 02/15/2019 |
| PROFILE 1014 | NCT01526928 | 3/11/2016 | 12/04/2014 | 05/16/2018 |
| PSMAfore | NCT04689828 | 3/28/2025 | 09/15/2024 | 06/09/2025 |
| RELATIVITY-047 | NCT03470922 | 3/21/2022 | 1/2022 | 5/2022 |
| SOPHIA | NCT02492711 | 12/16/2020 | 01/22/2021 | 11/04/2022 |
| SPARTAN | NCT01946204 | 2/14/2018 | 02/08/2018 | 08/11/2020 |
| SSGXVIII | NCT00116935 | 1/31/2012 | 03/28/2012 | 05/29/2020 |
| S-TRAC | NCT00375674 | 11/16/2017 | 10/10/2016 | 09/07/2017 |
| SUN 111 | NCT00428597 | 05/20/2011 | 02/10/2011 | 11/10/2016 |
| TITAN | NCT02489318 | 9/17/2019 | 07/04/2019 | 10/30/2023 |
| TRANSFORM | NCT03575351 | 6/24/2022 | 07/14/2022 | 04/06/2023 |
| TROPiCS-02 | NCT03901339 | 2/3/2023 | 08/26/2022 | 08/23/2023 |
| VISTA | NCT00111319 | 06/20/2008 | 08/28/2008 | 04/05/2010 |
| ZUMA-7 | NCT03391466 | 4/1/2022 | 12/11/2021 | 06/05/2023 |

# Table S5 Summary of the systematic search strategy

| Item | Details |
| --- | --- |
| Search objective | To identify phase Ⅱ–Ⅲ RCTs supporting US FDA oncology approvals (January 1, 2006 – September 1, 2025) with both initial (approval-stage) and updated safety publications. |
| Data sources | (1) FDA Hematology/Oncology (Cancer) Approvals Database; (2) MEDLINE (via PubMed); (3) ClinicalTrials.gov. |
| Search dates | Searches conducted between [start date] and September 1, 2025. |
| Source 1 — FDA Database | Browsed all hematology/oncology approval entries issued between Jan 1, 2006 and Sep 1, 2025; extracted drug name, indication, approval date, pivotal trial(s), and NCT identifier. Indications subsequently rescinded were removed. |
| Source 2 — MEDLINE/PubMed | Search string: ("[generic drug name]" OR "[brand name]") AND ("[approved indication / cancer type]") AND ("randomized" OR "randomised" OR "phase II" OR "phase III"). Field tags: Title/Abstract. Filters: English language; Humans; publication date 2006/01/01–2025/09/01. |
| Source 3 — ClinicalTrials.gov | Cross-referenced by NCT identifier obtained from FDA records and pivotal trial publications; retrieved trial registration, posted results, and citation links to identify all subsequent publications of the same trial. |
| Eligibility application | Two reviewers (WS and JZ) screened titles/abstracts in duplicate; full texts assessed against pre-specified eligibility criteria; discrepancies resolved by discussion. |
| Selection of updated evidence | When multiple post-approval publications existed for the same trial, the most recent publication (longest cumulative follow-up) was retained as the "updated evidence." |
| Records identified | 524 records identified; 329 RCTs met initial eligibility; 230 excluded for absence of an updated publication; 99 RCTs (106 pairwise comparisons after splitting 7 three-arm trials) included in the final analysis. |

# Table S6 Overall and Subgroup meta-analysis of grade 3+ adverse-event odds ratios (OR) before and after data updates

| Variable | Subgroup | No. of  studies | Pooled Initial OR  (95% CI) | Pooled Updated OR  (95% CI) | Studies with increased ORs | I² (initial reports) | I² (updated reports) |
| --- | --- | --- | --- | --- | --- | --- | --- |
| Overall | All Studies | 103 | 1.09 (0.93-1.22) | 1.29 (1.43-1.03) | 78 | 95.47 | 95.41 |
| Treatment_Line | First-line | 59 | 1.18 (0.95-1.27) | 1.46 (1.57-1.02) | 41 | 96.14 | 96.05 |
| Treatment_Line | Subsequent Line | 44 | 0.99 (0.77-1.15) | 1.27 (1.49-0.89) | 37 | 93.68 | 94.02 |
| Crossover | No | 63 | 1.06 (0.90-1.13) | 1.26 (1.35-0.94) | 46 | 92.8 | 93.17 |
| Crossover | Yes | 39 | 1.14 (0.82-1.38) | 1.59 (1.88-1.01) | 31 | 97.2 | 96.87 |
| Treatement comparisons | Targeted VS. Supportive care/Placebo | 7 | 1.74 (0.76-2.19) | 3.96 (4.68-1.02) | 6 | 96.02 | 95.26 |
| Treatement comparisons | Targeted VS. Targeted | 14 | 1.06 (0.82-1.22) | 1.36 (1.58-0.94) | 13 | 83.04 | 83.24 |
| Treatement comparisons | Hormonal VS. Hormonal | 4 | 1.24 (0.92-1.64) | 1.66 (1.97-1.36) | 4 | 82.54 | 58.71 |
| Treatement comparisons | Targeted VS. Chemotherapy | 7 | 1.21 (0.67-1.15) | 2.18 (2.23-0.59) | 5 | 88.79 | 89.62 |
| Treatement comparisons | Immunotherapy VS. Chemotherapy | 15 | 0.35 (0.26-0.42) | 0.49 (0.57-0.31) | 12 | 91.2 | 90.55 |
| Treatement comparisons | Immuno-Targeted VS. Targeted | 5 | 1.19 (1.02-1.48) | 1.40 (1.75-1.26) | 5 | 0 | 0 |
| Treatement comparisons | Immuno-Chemotherapy VS. Chemotherapy | 14 | 1.30 (1.14-1.26) | 1.48 (1.54-1.02) | 10 | 53.17 | 79.16 |
| Treatement comparisons | Immunotherapy VS. Supportive care/Placebo | 6 | 2.25 (1.41-2.28) | 3.59 (3.63-1.43) | 3 | 89.07 | 89.11 |
| Treatement comparisons | Others | 31 | 1.33 (0.93-1.47) | 1.89 (2.08-1.04) | 20 | 97 | 96.94 |
| Blinding | Yes | 39 | 1.33 (1.09-1.54) | 1.62 (1.91-1.25) | 31 | 91.67 | 92.62 |
| Blinding | No | 64 | 0.96 (0.76-1.05) | 1.22 (1.32-0.83) | 47 | 96.47 | 96.23 |
| phase | 3 | 101 | 1.10 (0.93-1.23) | 1.30 (1.45-1.04) | 76 | 95.54 | 95.48 |
| phase | 2 | 2 | 0.70 (0.47-0.80) | 1.03 (1.17-0.54) | 2 | 6.81 | 0 |
| Number of Patients | Large(>600) | 44 | 1.29 (1.00-1.39) | 1.67 (1.77-1.09) | 30 | 97.13 | 97.03 |
| Number of Patients | Medium (100-600) | 59 | 0.96 (0.78-1.10) | 1.17 (1.36-0.89) | 48 | 91.22 | 91.7 |
| FDA Approve Year | 2016–2020 | 54 | 0.90 (0.74-1.03) | 1.09 (1.27-0.83) | 43 | 93.75 | 94.79 |
| FDA Approve Year | Post-2020 | 34 | 1.45 (1.11-1.55) | 1.91 (2.01-1.19) | 22 | 95.13 | 94.74 |
| FDA Approve Year | Pre-2015 | 15 | 1.16 (0.71-1.31) | 1.90 (2.10-0.82) | 13 | 96.12 | 95.66 |
| Disease | Lung Cancer | 24 | 0.84 (0.62-0.99) | 1.15 (1.33-0.73) | 21 | 94.55 | 94.28 |
| Disease | Prostate Cancer | 8 | 1.33 (1.11-1.76) | 1.60 (2.10-1.48) | 7 | 69.75 | 68.66 |
| Disease | Others | 26 | 1.03 (0.72-1.09) | 1.46 (1.58-0.76) | 15 | 95.35 | 95.47 |
| Disease | Colorectal Cancer | 6 | 1.07 (0.62-1.25) | 1.86 (2.05-0.76) | 5 | 89.73 | 86.92 |
| Disease | Kidney Cancer | 9 | 1.51 (0.80-1.66) | 2.87 (3.13-0.88) | 7 | 96.74 | 96.76 |
| Disease | Multiple Myeloma | 5 | 1.91 (1.07-2.17) | 3.41 (4.11-1.15) | 5 | 86.93 | 87.93 |
| Disease | Melanoma | 12 | 0.87 (0.55-1.05) | 1.38 (1.73-0.64) | 11 | 92.31 | 93.21 |
| Disease | Breast Cancer | 13 | 1.44 (0.88-1.34) | 2.35 (2.22-0.81) | 7 | 97.03 | 97.17 |
| Metastatic | Yes | 85 | 1.02 (0.87-1.14) | 1.20 (1.35-0.96) | 64 | 94.09 | 94.15 |
| Metastatic | No | 18 | 1.50 (0.97-1.67) | 2.30 (2.50-1.11) | 14 | 97.12 | 96.85 |
| 5-year survival of indication | Low survival (<30%) | 33 | 1.03 (0.77-1.20) | 1.38 (1.59-0.90) | 28 | 95.35 | 95.19 |
| 5-year survival of indication | Intermediate (30%-69%) | 61 | 1.09 (0.92-1.19) | 1.30 (1.43-0.99) | 43 | 93.08 | 93.48 |
| 5-year survival of indication | High survival (70%-1) | 9 | 1.36 (0.62-1.47) | 2.98 (3.11-0.69) | 7 | 97.99 | 97.75 |
| Update time duration | Medium (12-24 months) | 36 | 1.08 (0.86-1.22) | 1.35 (1.50-0.99) | 27 | 92.17 | 90.93 |
| Update time duration | Short (<12 months) | 26 | 1.42 (1.09-1.58) | 1.85 (2.08-1.19) | 21 | 90.35 | 90.82 |
| Update time duration | Long (>24 months) | 41 | 0.95 (0.70-1.05) | 1.29 (1.43-0.77) | 30 | 97.38 | 97.41 |
| AE_Type_Group | All-cause | 46 | 1.29 (1.12-1.53) | 1.48 (1.77-1.32) | 38 | 85.03 | 85.53 |
| AE_Type_Group | Treatment-related | 57 | 0.95 (0.72-1.00) | 1.25 (1.31-0.77) | 40 | 97.11 | 97.01 |

Note, The grouping criteria for each subgroup are described in Table S2; Subgroups with fewer than two studies were excluded from quantitative analyses. Both the RMST ratio and OR represent comparisons of the experimental group versus the control group.

# Table S7 Overall and Subgroup Meta-analysis of serious adverse-event odds ratios (OR) before and after data updates

| Variable | Subgroup | No. of studies | Pooled Initial OR  (95% CI) | Pooled Updated OR  (95% CI) | Studies with increased ORs | I² (initial reports) | I² (updated reports) |
| --- | --- | --- | --- | --- | --- | --- | --- |
| Overall | All Studies | 70 | 1.32 (1.21-1.42) | 1.45 (1.59-1.27) | 49 | 71.65 | 81.14 |
| Treatment_Line | First-line | 40 | 1.40 (1.24-1.42) | 1.59 (1.67-1.21) | 26 | 75.69 | 86.99 |
| Treatment_Line | Subsequent Line | 30 | 1.20 (1.05-1.43) | 1.37 (1.62-1.26) | 23 | 57.72 | 55.49 |
| Crossover | No | 44 | 1.35 (1.20-1.34) | 1.53 (1.59-1.13) | 29 | 70.61 | 84.84 |
| Crossover | Yes | 25 | 1.26 (1.08-1.54) | 1.48 (1.76-1.35) | 19 | 74.75 | 67.56 |
| Treatement comparisons | Targeted VS. Supportive care/Placebo | 8 | 1.49 (0.97-1.57) | 2.28 (1.84-1.33) | 5 | 79.25 | 31.97 |
| Treatement comparisons | Targeted VS. Targeted | 7 | 0.97 (0.75-1.15) | 1.25 (1.54-0.85) | 7 | 59.36 | 68.61 |
| Treatement comparisons | Immunotherapy VS. Chemotherapy | 8 | 1.23 (0.84-1.30) | 1.81 (1.85-0.91) | 5 | 85.35 | 83.18 |
| Treatement comparisons | Targeted-Hormonal VS. Hormonal | 4 | 1.51 (1.06-1.85) | 2.15 (2.12-1.62) | 4 | 77.45 | 48.24 |
| Treatement comparisons | Immuno-Chemotherapy VS. Chemotherapy | 10 | 1.43 (1.14-1.47) | 1.79 (1.84-1.17) | 8 | 69.63 | 68.66 |
| Treatement comparisons | Targeted VS. Chemotherapy | 6 | 1.49 (1.07-1.46) | 2.08 (2.15-0.99) | 3 | 50.25 | 67.18 |
| Treatement comparisons | Others | 27 | 1.33 (1.19-1.41) | 1.50 (1.75-1.13) | 17 | 56.96 | 87.94 |
| Blinding | Yes | 27 | 1.44 (1.23-1.72) | 1.67 (1.93-1.53) | 19 | 72.48 | 56.52 |
| Blinding | No | 43 | 1.26 (1.12-1.24) | 1.42 (1.46-1.05) | 30 | 71.67 | 84.92 |
| Number of Patients | Large(>600) | 29 | 1.50 (1.32-1.49) | 1.71 (1.80-1.24) | 17 | 78.38 | 89.65 |
| Number of Patients | Medium (100-600) | 41 | 1.17 (1.04-1.36) | 1.32 (1.53-1.21) | 32 | 54.65 | 53.64 |
| FDA Approve Year | 2016–2020 | 33 | 1.24 (1.10-1.44) | 1.40 (1.64-1.27) | 27 | 68.26 | 71.97 |
| FDA Approve Year | Post-2020 | 27 | 1.43 (1.22-1.33) | 1.69 (1.71-1.03) | 15 | 67.74 | 88.03 |
| FDA Approve Year | Pre-2015 | 10 | 1.37 (1.05-1.62) | 1.78 (2.01-1.30) | 7 | 80.48 | 70.01 |
| Disease | Lung Cancer | 16 | 1.24 (1.01-1.36) | 1.52 (1.63-1.13) | 12 | 75.65 | 70.38 |
| Disease | Breast Cancer | 12 | 1.39 (1.09-1.52) | 1.78 (1.94-1.19) | 10 | 79.08 | 79.86 |
| Disease | Multiple Myeloma | 6 | 1.28 (0.96-1.54) | 1.71 (1.96-1.20) | 5 | 71.95 | 57.97 |
| Disease | Prostate Cancer | 6 | 1.21 (1.07-1.70) | 1.37 (2.03-1.42) | 6 | 0 | 52.55 |
| Disease | Others | 30 | 1.39 (1.18-1.35) | 1.64 (1.73-1.06) | 16 | 71.04 | 86.99 |
| Metastatic | Yes | 55 | 1.35 (1.22-1.40) | 1.49 (1.59-1.23) | 36 | 67.61 | 80.7 |
| Metastatic | No | 15 | 1.25 (0.99-1.49) | 1.57 (1.89-1.18) | 13 | 81.52 | 83.35 |
| 5-year survival of indication | Low survival (<30%) | 18 | 1.33 (1.07-1.42) | 1.64 (1.74-1.16) | 11 | 74.79 | 72.36 |
| 5-year survival of indication | Intermediate (30%-69%) | 43 | 1.37 (1.22-1.44) | 1.53 (1.68-1.24) | 31 | 69.03 | 83.62 |
| 5-year survival of indication | High survival (70%-) | 9 | 1.12 (0.85-1.30) | 1.47 (1.76-0.96) | 7 | 79.11 | 82.85 |
| Update time duration | Medium (12-24 months) | 29 | 1.30 (1.14-1.30) | 1.49 (1.62-1.05) | 20 | 68.02 | 87.21 |
| Update time duration | Short (<12 months) | 17 | 1.31 (1.07-1.38) | 1.61 (1.67-1.14) | 11 | 59.89 | 58.68 |
| Update time duration | Long (>24 months) | 24 | 1.36 (1.15-1.59) | 1.60 (1.84-1.38) | 18 | 79.9 | 75.48 |
| AE_Type_Group | All-cause | 34 | 1.27 (1.12-1.53) | 1.45 (1.73-1.36) | 26 | 72.7 | 69.85 |
| AE_Type_Group | Treatment-related | 36 | 1.38 (1.20-1.31) | 1.57 (1.59-1.07) | 23 | 68.56 | 86.2 |

Note, The grouping criteria for each subgroup are described in Table S2; Subgroups with fewer than two studies were excluded from quantitative analyses. Both the RMST ratio and OR represent comparisons of the experimental group versus the control group.

# Table S8 Overall and Subgroup Meta-analysis of ROR (Ratio of updated OR to initial OR) for grade 3+ AE

| Variable | Subgroup | No. of studies | ROR (95% CI) | p-value | I^2^ |
| --- | --- | --- | --- | --- | --- |
| Overall | All Studies | 103 | 1.095 (1.038–1.156) | 0.001 | 12.8 |
| Treatment_Line | First-line | 59 | 1.060 (0.989–1.135) | 0.099 | 19.9 |
| Treatment_Line | Subsequent Line | 44 | 1.177 (1.079–1.284) | 0.000 | 0 |
| Crossover | No | 63 | 1.029 (0.965–1.098) | 0.386 | 0 |
| Crossover | Yes | 39 | 1.203 (1.092–1.326) | 0.000 | 28.3 |
| Treatement comparisons | Targeted VS. Supportive care/Placebo | 7 | 1.171 (0.932–1.470) | 0.175 | 0 |
| Treatement comparisons | Targeted VS. Targeted | 14 | 1.125 (0.973–1.301) | 0.112 | 0 |
| Treatement comparisons | Hormonal VS. Hormonal | 4 | 1.310 (1.081–1.588) | 0.006 | 20.3 |
| Treatement comparisons | Targeted VS. Chemotherapy | 7 | 0.953 (0.720–1.262) | 0.735 | 0 |
| Treatement comparisons | Immunotherapy VS. Chemotherapy | 15 | 1.173 (0.990–1.389) | 0.065 | 34.2 |
| Treatement comparisons | Immuno-Targeted VS. Targeted | 31 | 1.045 (0.962–1.135) | 0.302 | 0 |
| Treatement comparisons | Immuno-Chemotherapy VS. Chemotherapy | 5 | 1.248 (0.993–1.569) | 0.058 | 0 |
| Treatement comparisons | Immunotherapy VS. Supportive care/Placebo | 14 | 0.974 (0.821–1.155) | 0.761 | 41.1 |
| Treatement comparisons | Others | 6 | 1.011 (0.823–1.242) | 0.918 | 0 |
| Blinding | Yes | 39 | 1.147 (1.042–1.263) | 0.005 | 26.8 |
| Blinding | No | 64 | 1.047 (0.985–1.114) | 0.142 | 0 |
| phase | 3 | 101 | 1.095 (1.037–1.156) | 0.001 | 13.3 |
| phase | 2 | 2 | 1.141 (0.657–1.981) | 0.639 | 0 |
| Number of Patients | Large(>600) | 44 | 1.072 (0.990–1.161) | 0.088 | 39.8 |
| Number of Patients | Medium (100-600) | 59 | 1.141 (1.049–1.242) | 0.002 | 0 |
| FDA Approve Year | 2016–2020 | 54 | 1.151 (1.076–1.232) | 0.000 | 0 |
| FDA Approve Year | Post-2020 | 34 | 1.037 (0.917–1.172) | 0.568 | 45.5 |
| FDA Approve Year | Pre-2015 | 15 | 1.097 (0.958–1.256) | 0.182 | 0 |
| Disease | Lung Cancer | 24 | 1.147 (1.033–1.274) | 0.010 | 4.7 |
| Disease | Prostate Cancer | 8 | 1.342 (1.170–1.539) | 0.000 | 2.2 |
| Disease | Others | 13 | 0.893 (0.753–1.060) | 0.195 | 45 |
| Disease | Colorectal Cancer | 26 | 1.034 (0.930–1.150) | 0.540 | 0 |
| Disease | Kidney Cancer | 6 | 1.106 (0.878–1.394) | 0.391 | 0 |
| Disease | Multiple Myeloma | 9 | 1.102 (0.941–1.291) | 0.230 | 0 |
| Disease | Melanoma | 5 | 1.105 (0.828–1.475) | 0.498 | 0 |
| Disease | Breast Cancer | 12 | 1.206 (1.009–1.443) | 0.040 | 0 |
| Metastatic | Yes | 85 | 1.102 (1.038–1.170) | 0.002 | 9.1 |
| Metastatic | No | 18 | 1.041 (0.944–1.148) | 0.419 | 0 |
| 5-year survival of indication | Low survival (<30%) | 33 | 1.141 (1.046–1.246) | 0.003 | 0 |
| 5-year survival of indication | Intermediate (30%-69%) | 61 | 1.085 (1.009–1.167) | 0.027 | 15.4 |
| 5-year survival of indication | High survival (70%-1) | 9 | 1.004 (0.845–1.194) | 0.961 | 16.5 |
| Update time duration | Medium (12-24 months) | 36 | 1.122 (1.030–1.223) | 0.009 | 0 |
| Update time duration | Short (<12 months) | 26 | 1.069 (0.953–1.199) | 0.253 | 0 |
| Update time duration | Long (>24 months) | 41 | 1.091 (0.998–1.193) | 0.057 | 33.7 |
| AE_Type_Group | All-cause | 46 | 1.157 (1.074–1.247) | 0.000 | 0 |
| AE_Type_Group | Treatment-related | 57 | 1.051 (0.971–1.137) | 0.220 | 24.8 |

Note, The grouping criteria for each subgroup are described in Table S2; Subgroups with fewer than two studies were excluded from quantitative analyses; OR represents comparisons of the experimental group versus the control group; PFS, and EFS refer to progression-free survival, and event-free (including disease-free or relapse-free) survival, respectively

# Table S9 Overall and Subgroup Meta-analysis of ROR (Ratio of updated OR to initial OR) for serious AE

| Variable | Subgroup | No. of studies | ROR (95% CI) | p-value | I^2^ |
| --- | --- | --- | --- | --- | --- |
| Overall | All Studies | 70 | 1.062 (0.965–1.170) | 0.219 | 46.8 |
| Treatment_Line | First-line | 40 | 0.999 (0.869–1.149) | 0.993 | 62.7 |
| Treatment_Line | Subsequent Line | 30 | 1.193 (1.064–1.338) | 0.003 | 0 |
| Crossover | No | 44 | 0.980 (0.853–1.126) | 0.771 | 53.9 |
| Crossover | Yes | 25 | 1.198 (1.081–1.328) | 0.001 | 2.5 |
| Treatement comparisons | Targeted VS. Supportive care/Placebo | 8 | 1.147 (0.902–1.460) | 0.264 | 0 |
| Treatement comparisons | Targeted VS. Targeted | 7 | 1.152 (0.914–1.452) | 0.230 | 0 |
| Treatement comparisons | Immunotherapy VS. Chemotherapy | 8 | 1.042 (0.856–1.267) | 0.684 | 0 |
| Treatement comparisons | Targeted-Hormonal VS. Hormonal | 4 | 1.173 (0.925–1.487) | 0.187 | 16.9 |
| Treatement comparisons | Immuno-Chemotherapy VS. Chemotherapy | 27 | 1.021 (0.820–1.272) | 0.851 | 75.2 |
| Treatement comparisons | Targeted VS. Chemotherapy | 10 | 1.029 (0.866–1.222) | 0.749 | 0 |
| Treatement comparisons | Others | 6 | 1.009 (0.768–1.325) | 0.951 | 0 |
| Blinding | Yes | 27 | 1.223 (1.101–1.358) | 0.000 | 0 |
| Blinding | No | 43 | 0.983 (0.856–1.129) | 0.808 | 56.8 |
| Number of Patients | Large(>600) | 29 | 0.979 (0.820–1.170) | 0.818 | 76.3 |
| Number of Patients | Medium (100-600) | 41 | 1.160 (1.039–1.294) | 0.008 | 0 |
| FDA Approve Year | 2016–2020 | 33 | 1.180 (1.075–1.296) | 0.001 | 0 |
| FDA Approve Year | Post-2020 | 27 | 0.891 (0.706–1.125) | 0.332 | 69.4 |
| FDA Approve Year | Pre-2015 | 10 | 1.151 (0.978–1.355) | 0.091 | 0 |
| Disease | Lung Cancer | 16 | 1.067 (0.930–1.224) | 0.355 | 0 |
| Disease | Breast Cancer | 30 | 0.933 (0.750–1.160) | 0.532 | 66.3 |
| Disease | Multiple Myeloma | 12 | 1.061 (0.917–1.227) | 0.430 | 0 |
| Disease | Prostate Cancer | 6 | 1.177 (0.950–1.459) | 0.137 | 0 |
| Disease | Others | 6 | 1.407 (1.188–1.666) | 0.000 | 0 |
| Metastatic | Yes | 55 | 1.025 (0.911–1.154) | 0.678 | 52.2 |
| Metastatic | No | 15 | 1.180 (1.039–1.340) | 0.011 | 0 |
| 5-year survival of indication | Low survival (<30%) | 18 | 1.047 (0.913–1.200) | 0.514 | 0 |
| 5-year survival of indication | Intermediate (30%-69%) | 43 | 1.041 (0.900–1.205) | 0.585 | 62.1 |
| 5-year survival of indication | High survival (70%-1) | 9 | 1.112 (0.947–1.305) | 0.196 | 0 |
| Update time duration | Medium (12-24 months) | 29 | 0.985 (0.808–1.202) | 0.883 | 68.4 |
| Update time duration | Short (<12 months) | 17 | 1.029 (0.873–1.212) | 0.737 | 0 |
| Update time duration | Long (>24 months) | 24 | 1.166 (1.058–1.285) | 0.002 | 0 |
| AE_Type_Group | All-cause | 34 | 1.206 (1.102–1.319) | 0.000 | 0 |
| AE_Type_Group | Treatment-related | 36 | 0.937 (0.793–1.109) | 0.450 | 60.2 |

Note, The grouping criteria for each subgroup are described in Table S2; Subgroups with fewer than two studies were excluded from quantitative analyses; OR represents comparisons of the experimental group versus the control group; PFS, and EFS refer to progression-free survival, and event-free (including disease-free or relapse-free) survival, respectively

# Table S10 Univariable meta-regression results of Grade 3+ AE by individual covariates

| Variable | Regression Coefficient | 95% CI Lower Limit | 95% CI Upper Limit | p‑Value |
| --- | --- | --- | --- | --- |
| Crossover | 0.12298 | 0.0232 | 0.22277 | 0.0157 |
| Treatment Line | 0.1214 | 0.01589 | 0.22691 | 0.0241 |
| AE Type | 0.11157 | 0.01162 | 0.21152 | 0.0287 |
| Blinding status | 0.09337 | -0.00899 | 0.19572 | 0.0738 |
| Updated Follow-up Time | -0.00211 | -0.00457 | 0.00035 | 0.0924 |
| Initial Follow-up Time | -0.00396 | -0.00906 | 0.00114 | 0.128 |
| Trial sample size | 0.07891 | -0.02499 | 0.18281 | 0.137 |
| Follow‑up duration | -0.00175 | -0.00472 | 0.00123 | 0.25 |
| Approve Year | -0.0102 | -0.02792 | 0.00753 | 0.26 |
| Metastatic setting | 0.05341 | -0.05974 | 0.16657 | 0.355 |
| 5-Year Survival Rate | -0.1122 | -1.14099 | 0.91659 | 0.831 |
| phase | -0.05231 | -0.60625 | 0.50163 | 0.853 |

Note, The grouping criteria for each subgroup are described in Table S1

# Table S11 Univariable meta-regression results of SAE by individual covariates

| Variable | Regression Coefficient | 95% CI Lower Limit | 95% CI Upper Limit | p‑Value |
| --- | --- | --- | --- | --- |
| 5-Year Survival Rate | -0.198 | -1.389 | 0.994 | 0.745 |
| AE Type | 0.268 | 0.133 | 0.403 | 0.000 |
| Approve Year | -0.027 | -0.048 | -0.006 | 0.011 |
| Blinding status | 0.230 | 0.091 | 0.369 | 0.001 |
| Crossover | 0.216 | 0.080 | 0.352 | 0.002 |
| Follow‑up duration | 0.005 | 0.000 | 0.009 | 0.050 |
| Metastatic setting | -0.146 | -0.296 | 0.004 | 0.057 |
| Trial sample size | 0.141 | 0.001 | 0.280 | 0.048 |
| phase | -0.031 | -0.733 | 0.670 | 0.930 |
| Updated Follow-up Time | 0.000 | -0.003 | 0.004 | 0.797 |
| Initial Follow-up Time | -0.008 | -0.015 | -0.001 | 0.017 |
| Treatment Line | 0.177 | 0.035 | 0.319 | 0.014 |

Note, The grouping criteria for each subgroup are described in Table S1

# Table S12 Multivariable meta-regression results of grade 3+AE

| Variable | Regression Coefficient | 95% CI Lower Limit | 95% CI Upper Limit | p Value |
| --- | --- | --- | --- | --- |
| Intercept | 0.009 | -0.146 | 0.163 | 0.911 |
| **Crossover** | **0.108** | **0.005** | **0.211** | **0.041** |
| AE Type | 0.099 | -0.007 | 0.204 | 0.066 |
| Treatment Line | 0.091 | -0.020 | 0.201 | 0.109 |
| Updated Follow‑up Time | -0.001 | -0.004 | 0.001 | 0.296 |
| Blinding status | 0.052 | -0.058 | 0.161 | 0.356 |

Note: All variables included in the regression model had variance inflation factors (VIFs) less than 5, indicating no multicollinearity; The model achieved an R^2^ of 0.494; The grouping criteria for each subgroup are provided in Table S1

# Table S13 Multivariable meta-regression results of SAE

| Variable | Regression Coefficient | 95% CI Lower Limit | 95% CI Upper Limit | p Value |
| --- | --- | --- | --- | --- |
| Intercept | -3.543 | -54.029 | 46.943 | 0.891 |
| **Initial Follow‑up Time** | **-0.013** | **-0.021** | **-0.005** | **0.002** |
| **Metastatic setting** | **-0.298** | **-0.492** | **-0.104** | **0.003** |
| **Blinding status** | **0.217** | **0.058** | **0.375** | **0.007** |
| **AE Type** | **0.220** | **0.052** | **0.388** | **0.010** |
| **Follow‑up duration** | **0.006** | **0.001** | **0.011** | **0.018** |
| **Trial sample size** | **0.176** | **0.021** | **0.330** | **0.026** |
| Treatment Line | 0.155 | -0.024 | 0.334 | 0.090 |
| Approve Year | 0.002 | -0.023 | 0.027 | 0.889 |
| Crossover | 0.009 | -0.154 | 0.171 | 0.914 |

Note: All variables included in the regression model had variance inflation factors (VIFs) less than 5, indicating no multicollinearity; The model achieved an R^2^ of 0.33; The grouping criteria for each subgroup are provided in Table S1

# Method S1 Toxicity Drift Predictor, Full Methods

Model. The TDP is a linear predictor of log(ROR), where ROR denotes the ratio of the updated to the initial odds ratio for serious adverse events. Coefficients (β₀–β₉) were taken directly from the SAE multivariable meta-regression of the main analysis; no re-fitting was performed. Equation is as follows:

Log(ROR)=0.497−0.013*Initial Follow-up Duration - 0.298*Metastatic +0.217*Open Label + 0.220*All Cause AE + 0.176*Large Sample+0.155*Later Line - 0.027*(Year-2020) +0.108*Crossover +0.006*Updated Time Horizon

ROR=exp(logROR); 95% prediction interval=exp(log ROR±1.96×0.18), where 0.18 is the residual SE on the log scale

Risk grades. Low (ROR < 1.00) | Mild (1.00–1.10) | Moderate (1.10–1.25) | High (≥ 1.25).

Inputs. Eight trial-level characteristics observable at FDA approval (initial follow-up duration in months; metastatic disease; blinding; AE reporting type; sample size; treatment line; approval year; cross-over) plus a user-specified post-approval re-evaluation horizon (months).

# Result S1. Variable coding and meta-regression coefficients

| Term | Symbol | Coefficient | Direction |
| --- | --- | --- | --- |
| Intercept | β_0_ | 0.497 | — |
| Initial follow-up (months) | β₁ | −0.013 | ↓ ROR |
| Metastatic (Yes = 1, No = 0) | β₂ | −0.298 | ↓ ROR |
| Blinding (Open-label = 1, Blinded = 0) | β₃ | 0.217 | ↑ ROR |
| AE type (All-cause = 1, TRAE = 0) | β₄ | 0.22 | ↑ ROR |
| Sample size (Large ≥ 600 = 1, Small = 0) | β₅ | 0.176 | ↑ ROR |
| Treatment line (Later = 1, First = 0) | β₆ | 0.155 | ↑ ROR |
| Approval year − 2020 | β₇ | −0.027 | ↓ ROR (newer drugs) |
| Cross-over allowed (Yes = 1, No = 0) | β₈ | 0.108 | ↑ ROR |
| Extended follow-up horizon (months) | β₉ | 0.006 | ↑ ROR |

Worked example

Hypothetical trial: open-label, large (≥ 600 patients), non-metastatic, later-line, all-cause AE reporting, no cross-over, 9-month initial follow-up, approved in 2022; Updated follow-up duration at a 24-month horizon.

| Term | Contribution to log-ROR |
| --- | --- |
| β₀ (intercept) | 0.497 |
| β₁ × Initial FU = −0.013 × 9 | −0.117 |
| β₂ × Metastatic = −0.298 × 0 | 0 |
| β₃ × Open-label = 0.217 × 1 | 0.217 |
| β₄ × All-cause = 0.220 × 1 | 0.22 |
| β₅ × Large sample = 0.176 × 1 | 0.176 |
| β₆ × Later-line = 0.155 × 1 | 0.155 |
| β₇ × (Year−2020) = −0.027 × 2 | −0.054 |
| β₈ × Cross-over = 0.108 × 0 | 0 |
| β₉ × Horizon = 0.006 × 24 | 0.144 |
| Sum = log(ROR) | 1.238 |
| ROR = exp(log-ROR) | 3.45 |

→ Predicted log-ROR = 1.238; ROR = 3.45 (95% PI 2.42–4.91); Grade = High
